# Cranberry Pomace Valorization: Composition, Extraction, Bioactivity, and Food Applications

**DOI:** 10.1111/1541-4337.70637

**Published:** 2026-09-10

**Authors:** Ali Ali Redha, Tuba Esatbeyoglu

**Affiliations:** ^1^ Department of Molecular Food Chemistry and Food Development, Institute of Food and One Health Gottfried Wilhelm Leibniz University Hannover Hannover Germany

**Keywords:** by‐product valorization, cranberry pomace, polyphenols, *Vaccinium macrocarpon*, *Vaccinium oxycoccos*

## Abstract

Cranberry pomace, the principal solid residue generated during cranberry processing, is increasingly recognized as a compositionally valuable coproduct rather than a waste material. This is particularly relevant in North America, where cranberry production is concentrated in the United States and Canada, the two leading producing countries. This review critically evaluates its chemical composition, recovery of valuable constituents, biological properties, and food‐related applications. Cranberry pomace retains substantial amounts of anthocyanins, flavonols, proanthocyanidins, and phenolic acids, together with a major dietary fiber fraction rich in structurally diverse polysaccharides and a smaller lipophilic fraction containing unsaturated fatty acids, tocochromanols, and triterpenoids. Ethanol‐based solvent extraction remains the most established practical baseline, whereas ultrasound‐assisted and pressurized liquid extraction offer improved recovery efficiency and shorter processing times. However, most extraction studies remain laboratory‐based, with limited pilot‐scale, environmental, and economic validation. Biological evidence is predominantly preclinical and is strongest for antioxidant, antimicrobial, and antiproliferative effects of phenolic‐rich fractions, while mechanistic evidence and human studies are scarce. Food applications include extruded and bakery products, fermented dairy‐type systems, and active or edible packaging, where cranberry pomace may provide fiber enrichment, natural color, and oxidative or microbial protection. Key priorities include standardized compositional reporting, gastrointestinal bioaccessibility studies, mechanistic and clinical validation, food‐grade scale‐up, and techno‐economic and environmental assessment. Cranberry pomace, therefore, represents a promising source of value‐added constituents. Still, wider utilization will depend on stronger evidence linking composition and processing to biological efficacy, product performance, and industrial feasibility.

**Abbreviations**
ABTS: 2,2′‐azino‐bis(3‐ethylbenzothiazoline‐6‐sulfonic acid)
AChE: acetylcholinesterase
AIS: alcohol‐insoluble solids
CC_50_
: 50% cytotoxic concentration
CE: catechin equivalents
CFU: colony‐forming units
CGE: cyanidin‐3‐*O*‐glucoside equivalents
COX‐1: cyclooxygenase‐1
COX‐2: cyclooxygenase‐2
DENV‐2: dengue virus serotype 2
DENV‐4: dengue virus serotype 4
DM: dry mass
DMAC: 4‐dimethylaminocinnamaldehyde
DP: degree of polymerization
DPPH: 2,2‐diphenyl‐1‐picrylhydrazyl
DW: dry weight
ECE: epicatechin equivalents
ECP: extruded cranberry pomace
EPPO: European and Mediterranean Plant Protection Organization
FRAP: ferric reducing antioxidant power
GAE: gallic acid equivalents
Gclc: glutamate–cysteine ligase catalytic subunit
GSH: reduced glutathione
HIUS: high‐intensity ultrasound
HO‐1: heme oxygenase‐1
HOMA‐IR: homeostatic model assessment of insulin resistance
HPLC: high‐performance liquid chromatography
HPMC: hydroxypropyl methylcellulose
IC_50_
: half‐maximal inhibitory concentration
II(QR): quinone reductase induction index
IL‐1β: interleukin‐1 beta
LH‐20: Sephadex LH‐20
LMH: chicken hepatoma cell line
LOX: lipoxygenase
LPS: lipopolysaccharide
MAE: microwave‐assisted extraction
MIC: minimum inhibitory concentration
MTS: 3‐(4,5‐dimethylthiazol‐2‐yl)‐5‐(3‐carboxymethoxyphenyl)‐2‐(4‐sulfophenyl)‐2*H*‐tetrazolium
NADES: natural deep eutectic solvents
NLRP3: NOD‐like receptor family pyrin domain‐containing protein 3
NM: not measured
NQO1: NAD(P)H:quinone oxidoreductase 1
Nrf2: nuclear factor erythroid 2‐related factor 2
ORAC: oxygen radical absorbance capacity
PA2E: procyanidin A2 equivalents
PAC: proanthocyanidin
PE: pomace extract/purified extract
PEF: pulsed electric field
PFE: pressurized fluid extraction
PLE: pressurized liquid extraction
PM: pomace material
PVPP: polyvinylpolypyrrolidone
QE: quercetin equivalents
QR: quinone reductase
ROS: reactive oxygen species
RSM: response surface methodology
SC‐CO_2_
: supercritical carbon dioxide
sEH: soluble epoxide hydrolase
SSF: solid‐state fermentation
TBARS: thiobarbituric acid reactive substances
TE: Trolox equivalents
TFA: trifluoroacetic acid
THP‐1: human monocytic leukemia cell line
TVN: total volatile nitrogen
Txn: thioredoxin
UAE: ultrasound‐assisted extraction
UCP: unextruded cranberry pomace
USDA: US Department of Agriculture
WST‐1: water‐soluble tetrazolium salt‐1
ZIKV: Zika virus

## 1 Introduction

Cranberry is the fruit of low‐growing evergreen shrubs or trailing vines of the genus *Vaccinium* in the family Ericaceae (Nemzer et al. 2022; Prasad et al. 2025). Among cranberry species, the two most relevant in food and agricultural contexts are the American cranberry, *Vaccinium macrocarpon*, and the European or small cranberry, *Vaccinium oxycoccos*. The American species is the principal commercial cranberry in North America, whereas *V. oxycoccos* occurs mainly in northern and central Europe and parts of Asia. In general, *V. macrocarpon* is characterized by larger fruits and greater commercial importance, while *V. oxycoccos* is more often associated with wild or semiwild habitats and regional use (Nemzer et al. 2022; Prasad et al. 2025). *V. macrocarpon* was domesticated less than 200 years ago, with commercial cultivation beginning in the 19th century through selections from native populations and later breeding programs producing modern cultivars with improved yield, fruit size, and anthocyanin content (Diaz‐Garcia et al. 2020). It now forms the basis of large‐scale cranberry cultivation and processing. In contrast, *V. oxycoccos* is not cultivated to any significant extent and is used predominantly through wild harvesting and regional processing (European and Mediterranean Plant Protection Organization [EPPO] 2026).

Cranberry fruit has attracted sustained scientific interest because of its distinctive composition, which includes organic acids, dietary fiber, vitamins, minerals, and a broad range of polyphenolic constituents, particularly anthocyanins, flavonols, phenolic acids, and proanthocyanidins (PACs) (Karim et al. 2023; Nemzer et al. 2022). Its composition is also influenced by cultivar and stage of maturity. In four American cranberry cultivars, namely, “Stevens,” “Pilgrim,” “Ben Lear,” and “Black Veil,” Viskelis et al. (2009) showed that total phenolics and anthocyanins changed markedly during ripening, with anthocyanins increasing strongly toward overripe stages. In contrast, the relative anthocyanin pattern remained broadly similar among cultivars. Cranberry is also widely recognized for its bioactive properties, particularly in relation to antioxidant, antimicrobial, and urinary tract‐associated effects, with broader evidence suggesting relevance to inflammation‐related and cardiometabolic outcomes as well (Jangid et al. 2025; Karim et al. 2023; Moro et al. 2024; Nemzer et al. 2022). Owing to this composition and functionality, cranberry is used predominantly in processed forms such as juice, juice blends, sauces, dried products, powders, and nutraceutical preparations rather than being consumed mainly as a fresh fruit (Karim et al. 2023; Nemzer et al. 2022).

The industrial significance of cranberries is overwhelmingly concentrated in North America, and this has direct implications for by‐product generation. According to US Department of Agriculture (USDA) data, the United States produces approximately two‐thirds of the world's cranberries, and the US crop will reach 8.95 million barrels in 2024, with production concentrated mainly in Wisconsin, Massachusetts, Oregon, and New Jersey (USDA Economic Research Service 2025). Canada is the second‐largest producer after the United States; in 2024, it produced 211,790 metric tons of cranberries, with Quebec and British Columbia together accounting for almost 95% of the national crop and Quebec alone contributing 154,288 metric tons, or almost 73% of Canadian production (Agriculture and Agri‐Food Canada 2025). Cranberry also occupied 8832 ha in Canada in 2024. Because only a limited proportion of cranberries are marketed fresh, whereas the majority are processed into juices, sauces, dried fruits, and related ingredients, industrial production inevitably results in substantial quantities of pomace. In this respect, cranberry processing presents both a challenge and an opportunity. If insufficiently utilized, pomace contributes to biomass disposal and associated environmental burdens; however, within a sustainable‐development and circular‐bioeconomy framework, the same material can be viewed as a concentrated stream of recoverable phytochemicals, structural carbohydrates, and seed‐associated constituents suitable for value‐added use (Karim et al. 2023; Nemzer et al. 2022).

**FIGURE 1:**
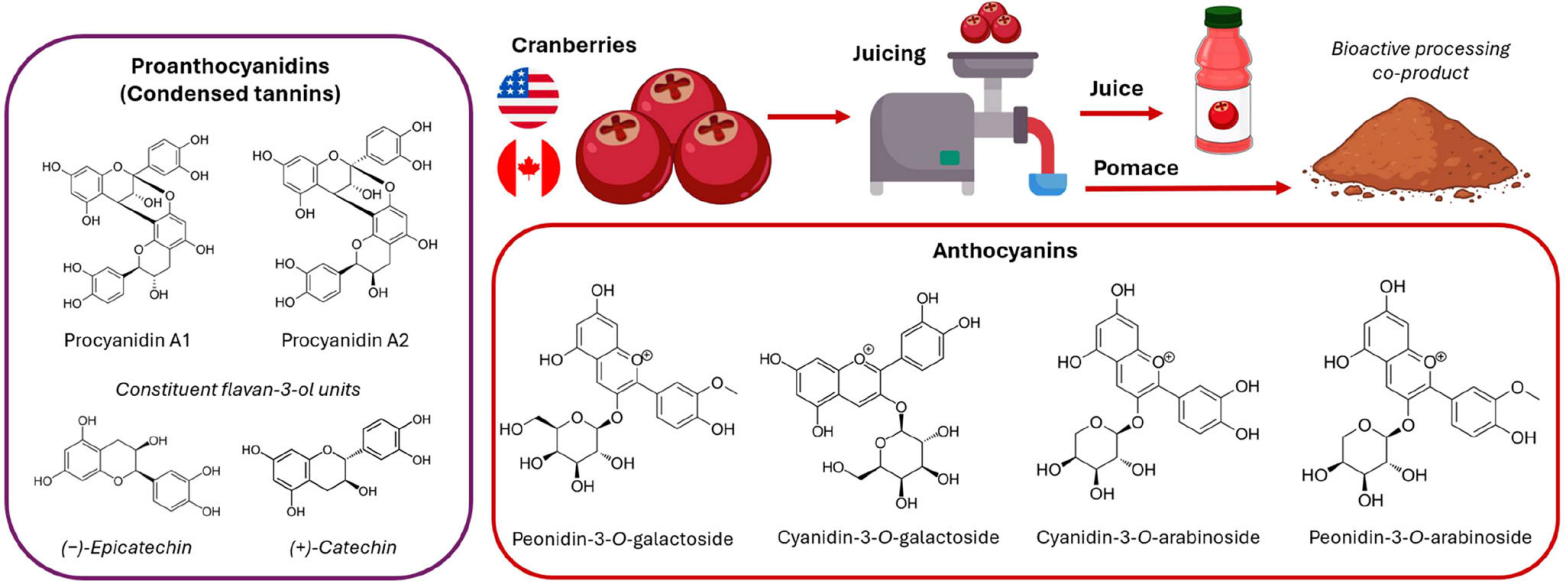
Formation of cranberry pomace during juice processing and representative polyphenolic constituents retained in the pomace matrix.

Cranberry pomace consists mainly of peel, seeds, pulp residues, and minor stem material remaining after juice extraction, and its composition reflects both the intrinsic chemistry of the fruit and the partitioning of constituents during processing (Figure 1). Earlier work has shown that pomace retains a substantial proportion of fruit polyphenols, whereas sugars and organic acids are transferred more extensively to the juice fraction (Oszmiański et al. 2015). In general terms, cranberry pomace is characterized by high dietary fiber, appreciable residual phenolics, and a seed‐associated lipophilic fraction, together with smaller amounts of protein, minerals, and residual soluble solids (White et al. 2010b; Piasecka et al. 2023; Spadoni Andreani et al. 2021a; Ross et al. 2017). These retained constituents are important because the biological activities long associated with cranberry fruit, particularly antioxidant, antimicrobial, and urinary tract‐related effects, are closely linked to the same classes of compounds that remain concentrated in the pomace, especially polyphenols and PACs (Figure 1) (Jangid et al. 2025; Karim et al. 2023; Moro et al. 2024; Nemzer et al. 2022).

Despite the extensive literature on cranberry phytochemicals and biological activity, cranberry pomace should not be regarded simply as a residual form of the whole fruit. Juice processing substantially modifies the cranberry matrix: readily soluble constituents, particularly sugars and organic acids, are preferentially transferred to the juice, whereas much of the skin‐, seed‐, and cell wall‐associated phenolic fraction remains in the pomace (Oszmiański et al. 2015). Anthocyanins are distributed between both streams, while polymeric and less readily extractable PACs, particularly A‐types, become comparatively more prominent in the residual matrix with likely implications for extractability, bioaccessibility, biological activity, and technological functionality. These properties cannot therefore be inferred directly from studies on cranberry fruit or juice. Against this background, the present review evaluates cranberry pomace as a distinct processing co‐product, rather than as a residual form of the whole fruit. To provide a comprehensive assessment, Web of Science, Scopus, PubMed, and Google Scholar were searched for relevant publications up to March 2026, with the search updated in July 2026. Search terms included “cranberry pomace,” “cranberry press cake,” “cranberry press residue,” “*Vaccinium macrocarpon* pomace,” and “*Vaccinium oxycoccos* pomace.” Studies examining cranberry pomace or derived fractions were screened and grouped by composition, stabilization, extraction, biological activity, and food or packaging applications. Evidence was critically compared within each category, while studies on cranberry fruit, juice, or other berry pomaces were included only for relevant comparative or mechanistic context. This review therefore evaluates cranberry pomace as a distinct processing coproduct.

## 2 Composition of Cranberry Pomace

Cranberry pomace is a heterogeneous matrix composed mainly of peel, seeds, residual pulp, and occasional stem material. Published proximate data (Bajerska et al. 2018; Jureviciute et al. 2022; White et al. 2010b) relate predominantly to dried pomace powders, which contain approximately 4.5–5.6 g/100 g residual moisture. Reported values for dried cranberry pomace indicate that dietary fiber constitutes approximately 71.2–73.0 g/100 g, comprising 59.9–67.3 g/100 g insoluble fiber and 5.7–12.7 g/100 g soluble fiber. Protein, lipid, ash, and residual nonfiber carbohydrates were reported at approximately 2.0–7.4, 9.8–12.0, 0.96–1.1, and 8.4–9.1 g/100 g dry mass (DM), respectively. Reported total phenolic values vary more widely with sample preparation, extraction, assay, and expression basis, ranging from approximately 3.9 to 24.9 mg gallic acid equivalents (GAE)/g DM (Table 1). The pomace also retains a seed‐ and cuticle‐associated lipophilic fraction containing unsaturated fatty acids, triterpenoids, phytosterols, carotenoids, and vitamin E homologs (Piasecka et al. 2023; Tamkutė, Liepuoniūtė, et al. 2020; Tamkutė, Pukalskas, et al. 2020). In contrast, individual minerals and vitamins have not been characterized sufficiently to define representative concentration ranges. The following subsections therefore focus on the three most extensively investigated fractions: phenolics, polysaccharides and dietary fiber, and lipids and triterpenoid constituents.

**TABLE 1:** Phenolic composition of cranberry pomace and pomace‐derived extracts reported in the literature.

Study	Pomace sample	Pretreatment	Extraction	Phenolic content	Tannin content	Anthocyanin content	Individual polyphenols
Alrugaibah et al. (2021)	Cranberry—USA	Freeze drying	*HIUS‐assisted extraction*: 0.25 g of cranberry pomace powder was extracted with 10 mL of NADES or 75% ethanol (1:40 w/v) using high‐intensity ultrasound at 100% amplitude in a water bath at 60°C for 30 min (2 × 15 min rounds), followed by centrifugation at 3260 × *g* to obtain a clear supernatant for analysis	NM	9.93–32.46 mg ECE/g DM (DMAC assay)	0.59–1.54 CGE/g DM	Peonidin‐3‐*O*‐arabinoside = 19–60 mg/100 g DM Peonidin‐3‐*O*‐galactoside = 22–55 mg/100 g DM Cyanidin‐3‐*O*‐galactoside = 4–30 mg/100 g DM Cyanidin‐3‐*O*‐arabinoside = 4–19 mg/100 g DM Peonidin‐3‐*O*‐glucoside = 1–11 mg/100 g DM
Ross et al. (2020)	Cranberry—Canada	Freeze drying or hot air drying (50°C–70°C)	*Solvent extraction*: Dried pomace (1 g) was extracted with 70% methanol (1:20, w/v) for 30 min with shaking, followed by centrifugation and filtration	13.54–15.17 mg GAE/g DM Total flavonols: 2.86–3.03 mg QE/g DM	NM	1.07–1.47 mg CGE/g DM	
Franco‐Londoño et al. (2025)	Cranberry—Canada	Freeze drying	*HIUS‐assisted extraction*: ∼0.5 g of ground cranberry pomace was extracted with NADES, ethanol, or water using high‐intensity ultrasound at 600 W for 5 min, with the extraction vessel immersed in an ice bath to minimize thermal degradation, followed by centrifugation at 7155 × *g* for 30 min	1.93–22.29 mg GAE/g DM	NM	0.04–0.86 mg CGE/g DM	
Klavins et al. (2017)	Cranberry (*Vaccinium macrocarpon*)—Jūrmala City, Latvia	Freeze drying	*Solvent extraction (Soxhlet, MAE, UAE, and maceration)*: Powder was extracted using four methods. *Soxhlet*: 1.0 g with 100 mL of 96% ethanol containing 0.5% TFA at 80°C for 12 h. *MAE*: 0.5 g with 50 mL of solvent at 600 W, heated to 80°C in 10 min and held for 20 min. *UAE*: 0.5 g with 50 mL of solvent	8.9–48.0 mg GAE/g DM	NM	0.98–4.51 mg CGE/g DM	
			using 100 or 360 W ultrasound below 30°C, followed by 24 h shaking in the dark. *Maceration*: 0.5 g with 50 mL of solvent by shaking in the dark for 24 h				
Tamkutė et al. (2020)	Cranberry—Lithuania	Hot air drying (35°C)	*PLE‐EtOH*: Pomace (after SC‐CO_2_ defatting) was extracted by pressurized liquid extraction with ethanol using three static cycles at 10.3 MPa, with extraction temperature 50°C–90°C and static time 5–15 min per cycle	14.03–22.15 mg GAE/g DM 28.49–43.32 mg GAE/g DM PE	74.87–113.7 mg PAC/g DM 157.6–225.2 mg PAC/g DM PE	0.97–1.91 mg CGE/g DM 2.04–3.81 mg CGE/g DM PE	
White et al. (2010b)	Cranberry—USA	Drying (method not specified)	*Solvent extraction*: Triple extraction with acetone/water/acetic acid (70:29.5:0.5, v/v/v), followed by Sephadex LH‐20 cleanup and 70% aqueous acetone elution of procyanidins	NM	NM	NM	*Procyanidins*: Procyanidin A‐type dimers (DP2) = 82.6 ± 2.3 mg/100 g DM Procyanidin A‐type trimers (DP3) = 30.8 ± 2.5 mg/100 g DM Procyanidin B‐type tetramers (DP4) = 22.9 ± 3.6 mg/100 g DM Procyanidin A‐type hexamers (DP6) = 12.1 ± 1.3 mg/100 g DM Procyanidin A‐type pentamers (DP5) = 7.1 ± 1.2 mg/100 g DM Procyanidin monomers (DP1; catechin/epicatechin) = 5.8 ± 0.4 mg/100 g DM Procyanidin B‐type dimers (DP2) = 4.4 ± 0.7 mg/100 g DM Procyanidin B‐type trimers (DP3) = 1.6 ± 0.4 mg/100 g DM *Anthocyanins*: Cyanidin‐3‐*O*‐arabinoside = 49.6 ± 6.8 mg/100 g DM Peonidin‐3‐*O*‐arabinoside = 26.6 ± 0.5 mg/100 g DM Peonidin‐3‐*O*‐galactoside = 20.1 ± 0.5 mg/100 g DM Cyanidin‐3‐*O*‐galactoside = 13.2 ± 0.2 mg/100 g DM
							Peonidin‐3‐*O*‐glucoside = 7.4 ± 0.3 mg/100 g DM Cyanidin‐3‐*O*‐glucoside = 4.5 ± 0.2 mg/100 g DM *Flavonols*: Quercetin = 146.2 ± 22.7 mg/100 g DM Myricetin = 55.6 ± 2.6 mg/100 g DM Quercetin‐3‐*O*‐benzoyl galactoside = 27.5 ± 3.4 mg/100 g DM Quercetin‐3‐*O*‐rhamnoside = 18.5 ± 3.4 mg/100 g DM Quercetin‐3‐*O*‐arabinofuranoside = 16.7 ± 3.5 mg/100 g DM Quercetin‐3‐*O*‐arabinopyranoside = 15.2 ± 3.6 mg/100 g DM Quercetin‐3‐*O*‐galactoside = 12.8 ± 3.6 mg/100 g DM Methoxyquercetin‐3‐*O*‐xyloside = 11.4 ± 3.7 mg/100 g DM Quercetin‐3‐*O*‐xyloside = 5.5 ± 0.3 mg/100 g DM Quercetin‐3‐*O*‐coumaroyl galactoside = 2.3 ± 0.3 mg/100 g DM Myricetin‐3‐*O*‐arabinoside = 1.8 ± 0.1 mg/100 g DM Myricetin‐3‐*O*‐xyloside = 1.5 ± 0.3 mg/100 g DM
Oszmiański et al. (2015)	Cranberry (*V. macrocarpon*)—Wola Mysłowska, Poland	Freeze drying	*UAE*: Powdered pomace (2 g) was extracted twice with 50 mL methanol containing 2.0% formic acid under sonication for 20 min, with occasional shaking, followed by centrifugation and membrane filtration before analysis	NM	NM	NM	*Flavan‐3‐ols and procyanidins*: Procyanidin polymers = 2360.11 mg/100 g DM A‐type procyanidin trimer = 119.68 mg/100 g DM (−)‐Epicatechin = 73.56 mg/100 g DM A‐type procyanidin dimer (Isomer 1) = 64.67 mg/100 g DM A‐type procyanidin dimer (Isomer 2) = 44.34 mg/100 g DM B‐type procyanidin dimer = 43.31 mg/100 g DM (+)‐Catechin = 28.98 mg/100 g DM
							*Anthocyanins*: Peonidin‐3‐*O*‐galactoside = 4729.53 mg/100 g DM Cyanidin‐3‐*O*‐galactoside = 3405.57 mg/100 g DM Cyanidin‐3‐*O*‐arabinoside = 2320.81 mg/100 g DM Peonidin‐3‐*O*‐arabinoside = 1900.02 mg/100 g DM Peonidin‐3‐*O*‐glucoside = 465.24 mg/100 g DM Cyanidin‐3‐*O*‐glucoside = 115.63 mg/100 g DM Delphinidin‐3‐*O*‐glucoside = 41.81 mg/100 g DM Malvidin‐3‐*O*‐arabinoside = 15.92 mg/100 g DM *Flavonols* Myricetin‐3‐*O*‐pentoside (isomer 1) = 1018.21 mg/100 g DM Myricetin‐3‐*O*‐pentoside (isomer 2) = 628.62 mg/100 g DM Quercetin‐3‐*O*‐rhamnoside = 217.04 mg/100 g DM Myricetin‐3‐*O*‐pentoside (isomer 3) = 192.18 mg/100 g DM Methoxyquercetin‐hexoside (isomer 1) = 97.34 mg/100 g DM Quercetin‐3‐*O*‐pentoside (isomer 1) = 70.02 mg/100 g DM Quercetin‐3‐*O*‐pentoside (isomer 2) = 64.79 mg/100 g DM Quercetin‐3‐*O*‐galactoside = 63.50 mg/100 g DM Quercetin‐3‐*O*‐pentoside (isomer 3) = 61.06 mg/100 g DM Myricetin‐3‐*O*‐pentoside (isomer 4) = 44.91 mg/100 g DM
							Methoxyquercetin‐hexoside (isomer 2) = 24.78 mg/100 g DM Methoxyquercetin‐pentoside (isomer 1) = 18.11 mg/100 g DM Methoxyquercetin‐pentoside (isomer 2) = 16.04 mg/100 g DM Quercetin‐benzoyl‐galactoside = 10.15 mg/100 g DM Quercetin‐*p*‐coumaroyl‐hexoside = 6.03 mg/100 g DM *Phenolic acids*: *p*‐Coumaroyl‐hexose isomer 1 = 165.16 mg/100 g DM Caffeoyl‐hexoside isomer = 74.59 mg/100 g DM Sinapoyl‐hexose = 56.54 mg/100 g DM Caffeoyl‐hexoside = 19.72 mg/100 g DM Chlorogenic acid = 17.14 mg/100 g DM *p*‐Coumaroyl‐hexose isomer 2 = 13.40 mg/100 g DM Caffeoyl‐dihexoside = 5.55 mg/100 g DM *p*‐Coumaroyl‐hexose isomer 3 = 2.18 mg/100 g DM *p*‐Coumaroyl‐hexose = 2.14 mg/100 g DM
Kühn and Temelli (2017)	Cranberry—Germany	Hot air drying (40°C)	*Solvent extraction*: Pomace (2 g) was extracted with 70% acidified aqueous ethanol (70:29.5:0.5 ethanol:water:acetic acid, v/v/v) for 4 h at 22°C with stirring	19.78 ± 0.32 mg GAE/g DM	NM	NM	Peonidin‐3‐*O*‐galactoside = 86.4 ± 0.2 mg/100 g DM Cyanidin‐3‐*O*‐arabinoside = 49.5 ± 0.1 mg/100 g DM Peonidin‐3‐*O*‐arabinoside = 47.2 ± 0.3 mg/100 g DM Cyanidin‐3‐*O*‐galactoside = 44.5 ± 0.5 mg/100 g DM Malvidin‐3‐*O*‐galactoside = 12.3 ± 0.3 mg/100 g DM Malvidin‐3‐*O*‐arabinoside = 7.5 ± 0.2 mg/100 g DM Peonidin‐3‐*O*‐glucoside = 7.4 ± 0.2 mg/100 g DM
White et al. (2010c)	Cranberry—USA	Drying (method not specified)	*Alkaline hydrolysis followed by solvent extraction*: Ground pomace (0.5 g) was treated with 5 mL of 2–6 N NaOH under nitrogen at 25°C, 40°C, or 60°C for 5 min to 24 h, then neutralized to pH 6–7 with 4 N HCl. After delipidation with hexane, procyanidin monomers to trimers were extracted twice with ethyl acetate, whereas monomers through polymers were extracted separately with acetone/water/acetic acid (70:29.5:0.5, v/v/v) by Ultra‐Turrax homogenization, repeated three times and pooled	NM	NM	NM	Procyanidin monomers (DP1) = 44.6–85.5 mg/100 g DM Procyanidin dimers (DP2) = 248.9–519.3 mg/100 g DM Procyanidin trimers (DP3) = 85.6–144.5 mg/100 g DM
Ross et al. (2020)	Organic cranberry (*V. macrocarpon*)—Quebec, Canada	Freeze drying	*Solvent extraction*: Pomace (2.5 g) was extracted three times with 70% aqueous methanol using 25 mL for the first extraction and 12.5 mL for each of the second and third extractions, with shaking for 60 min, followed by centrifugation and filtration	24.87 ± 0.66 mg GAE/g DM (Folin–Ciocalteu method) 12.99 ± 0.29 mg GAE/g DM (Glories method) Total flavonols: 3.08 ± 0.06 mg QE/g DM	21.86 ± 0.68 mg GAE/g DM (PVPP precipitation/Folin difference method)	4.46 ± 0.17 mg CGE/g DM	Peonidin‐3‐*O*‐galactoside = 158 ± 16 mg/100 g DM Cyanidin‐3‐*O*‐galactoside = 120 ± 13 mg/100 g DM Cyanidin‐3‐*O*‐arabinoside = 85 ± 9 mg/100 g DM Peonidin‐3‐*O*‐arabinoside (isomer 1) = 68 ± 7 mg/100 g DM Peonidin‐3‐*O*‐glucoside (isomer 1) = 17 ± 2 mg/100 g DM Malvidin‐3‐*O*‐arabinoside = 6 ± 1 mg/100 g DM Cyanidin‐3‐*O*‐arabinoside/Delphinidin‐3‐*O*‐glucoside = 4 ± 1 mg/100 g DM Delphinidin‐3‐*O*‐galactoside = 2 ± 0 mg/100 g DM Peonidin‐3‐*O*‐glucoside (isomer 2) = 2 ± 0 mg/100 g DM Peonidin‐3‐*O*‐arabinoside (isomer 2) = 2 ± 0 mg/100 g DM
			*Solvent extraction*: Pomace (12 kg) was extracted with 80% aqueous ethanol at a 1:5 (w/v) ratio for 1 h, then filtered, concentrated, freeze‐dried, and cryomilled; the dried extractives were subsequently extracted three times with 70% aqueous methanol (2.5 g sample; 25 mL, then 12.5 and 12.5 mL) before analysis	54.35 ± 0.85 mg GAE/g DM (Folin–Ciocalteu method) 36.25 ± 1.39 mg GAE/g DM (Glories method) Total flavonols: 11.74 ± 0.48 mg QE/g DM	48.09 ± 1.26 mg GAE/g DM (PVPP precipitation/Folin difference method)	11.14 ± 0.39 mg CGE/g DM	Peonidin‐3‐*O*‐galactoside = 275 ± 4 mg/100 g DM Cyanidin‐3‐*O*‐galactoside = 205 ± 4 mg/100 g DM Cyanidin‐3‐*O*‐arabinoside = 147 ± 3 mg/100 g DM Peonidin‐3‐*O*‐arabinoside (isomer 1) = 114 ± 2 mg/100 g DM Peonidin‐3‐*O*‐glucoside (isomer 1) = 50 ± 0 mg/100 g DM Peonidin‐3‐*O*‐glucoside (isomer 2) = 20 ± 0 mg/100 g DM Peonidin‐3‐*O*‐arabinoside (isomer 2) = 15 ± 0 mg/100 g DM Malvidin‐3‐*O*‐arabinoside = 13 ± 0 mg/100 g DM Cyanidin‐3‐*O*‐arabinoside/Delphinidin‐3‐*O*‐glucoside = 10 ± 0 mg/100 g DM Malvidin‐3‐*O*‐glucoside = 9 ± 0 mg/100 g DM Peonidin‐3‐*O*‐glucoside (isomer 3) = 7 ± 0 mg/100 g DM Delphinidin‐3‐*O*‐galactoside = 4 ± 0 mg/100 g DM
Klavins et al. (2018)	Cranberry (*V. macrocarpon*)—Jūrmala City, Latvia	Hot air drying (40°C)	*UAE*: 0.5 g of dried, homogenized American cranberry press residue was extracted with 50 mL of aqueous methanol or ethanol acidified with TFA or formic acid using a 100 W ultrasound bath, followed by shaking in the dark for 24 h and filtration	32–35 mg GAE/g DM PE	NM	7.08 ± 0.97 mg CGE/g DM PE	
Harrison et al. (2013)	Cranberry—Canada	—	*Ethanolic extraction*: Cranberry press cake was extracted with 95% ethanol using either repeated 24 h solvent soaking with decanting and final bladder	Total phenolic acids: 120.8–142.3 mg CE/g DM PE Total flavonols: 8.76–11.24 mg QE/g DM PE	NM	9.89–19.24 mg CGE/g DM PE	
			pressing, or a 44 h soak followed by decanting and immediate bladder pressing. The extracts were then freeze‐dried and milled before analysis				
Roopchand et al. (2013)	Cranberry—USA	—	*Hot solvent extraction*: Frozen depectinized pomace was extracted with 50% or 75% aqueous ethanol at 5:1 or 10:1 solvent:wet pomace ratios, adjusted to pH 2, and heated at 80°C for 2 h with continuous agitation	118–157 mg GAE/g DM PE	73–96 mg procyanidin A2 equivalents PA2E/g (chromatographic analysis)	NM	
Saldaña et al. (2021)	Cranberry—Canada	Freeze drying	*PFE*: Milled pomace (2 g) was extracted with pressurized water, 30%–100% ethanol, or 5% citric acid at 40°C–160°C and 50 or 200 bar (5 mL/min), and extracts were collected over 5–30 min	84.96 ± 7.82 mg GAE/g DM	NM	6.02–8.42 mg CGE/g DM	Cyanidin‐3‐*O*‐galactoside = 81–370 mg/100 g DM PE Cyanidin‐3‐*O*‐arabinoside = 27–76 mg/100 g DM PE Cyanidin‐3‐*O*‐glucoside = 23–46 mg/100 g DM PE Peonidin‐3‐*O*‐arabinoside = 16–44 mg/100 g DM PE Peonidin‐3‐*O*‐galactoside = 15–34 mg/100 g DM PE Peonidin‐3‐*O*‐glucoside = 10–19 mg/100 g DM PE
Brzezowska et al. (2024)	Cranberry (*V. macrocarpon*)—Rokietnica, Poland	Freeze drying or vacuum drying (60°C or 90°C)	*Solvent extraction followed by resin purification*: Defrosted cranberry pomace (∼15 kg) was extracted with 30% acetone, 50% ethanol, or acidified 50% ethanol (pH 2) at a pomace‐to‐solvent ratio of 1:4 (w/v), with sonication for 15 min at the start and again after 24 h, followed by filtration. The solvent was removed under vacuum, and the resulting extract (∼6 L per solvent) was purified on XAD‐16 Amberlite resin	NM	NM	NM	Flavonols = 8990–12,610 mg/100 g PE Flavan‐3‐ols = 2050–3450 mg/100 g PE Phenolic acids = 1940–2430 mg/100 g PE Anthocyanins = 1380–2280 mg/100 g PE (authors quantified these phenolic groups chromatographically, but did not report the full individual compound breakdown)

*Note*: Cranberry materials in the literature may refer to different species, most commonly *V. macrocarpon* and *Vaccinium oxycoccos*. For studies from the United States and Canada, the source would generally be expected to be *V. macrocarpon*; however, when species identity was not explicitly stated in the sample description or methods, it is reported in this table only as cranberry, followed by the country of origin.

Abbreviations: CE, catechin equivalents; CGE, cyanidin‐3‐glucoside equivalents; DM, dry mass; DMAC, 4‐dimethylaminocinnamaldehyde; DP, degree of polymerization; ECE, epicatechin equivalents; GAE, gallic acid equivalents; HIUS, high‐intensity ultrasound; LH‐20, Sephadex LH‐20; MAE, microwave‐assisted extraction; NADES, natural deep eutectic solvents; NM, not measured; PA2E, procyanidin A2 equivalents; PAC, proanthocyanidins; PE, pomace extract/purified extract; PFE, pressurized fluid extraction; PLE, pressurized liquid extraction; PM, pomace material; PVPP, polyvinylpolypyrrolidone; QE, quercetin equivalents; RSM, response surface methodology; SC‐CO_2_, supercritical carbon dioxide; Soxhlet, Soxhlet extraction; TFA, trifluoroacetic acid; UAE, ultrasound‐assisted extraction; w/v, weight/volume.

### 2.1 Phenolics

Cranberry pomace is a phenolic‐rich by‐product because a substantial proportion of the fruit polyphenols remain associated with the skin‐ and seed‐rich residue after juice processing. As shown in Table 1, the available literature consistently identifies four main phenolic groups in cranberry pomace, namely, anthocyanins, flavonols, flavan‐3‐ols/PACs, and phenolic acids. However, the reported absolute concentrations vary markedly with raw material, processing history, and the analytical basis used for expression. On a pomace DM basis, the total phenolic content ranges from approximately 8.9 to 24.9 mg GAE/g DM in several pomace samples. At the same time substantially higher values have also been reported for some processed or pressurized fluid‐derived materials. As also summarized in Table 1, total anthocyanins in pomace range from 0.04 to 0.86 mg cyanidin‐3‐glucoside equivalents (CGE)/g DM in some samples to 4.46 mg CGE/g DM in conventionally extracted pomace and up to 11.14 mg CGE/g DM in pomace‐derived extractives. Tannin‐ or PAC‐related values, although reported using different assays and standards, also span a wide range, including 9.93–32.46 mg epicatechin equivalents (ECE)/g DM, 21.86 mg GAE/g DM, 73–96 mg procyanidin A2 equivalents (PA2E)/g, and 157.6–225.2 mg PAC/g DM in pomace‐derived extracts. Overall, the data compiled in Table 1 indicate that cranberry pomace should be regarded not as a residual material depleted of phytochemicals, but as a compositionally complex phenolic matrix in which anthocyanins, flavonols, and especially PACs are retained to a substantial extent after pressing.

The composition of cranberry pomace is also influenced by the state of the fruit before pressing. Oszmiański et al. (2015) showed that pomace obtained from whole fruits retained a larger proportion of total fruit polyphenols than pomace obtained after crushing, with 95.4% and 78.9% of the fruit polyphenols remaining in pomace, respectively. In contrast, crushing increased juice yield from 53.0% to 77.6% and reduced pomace yield from 47.0% to 22.4%, while also promoting the transfer of sugars and acids into the juice fraction. These observations are compositionally important because they indicate that phenolics, being concentrated largely in the structural fruit tissues, are retained in pomace to a much greater extent than the more water‐soluble sugars and organic acids, but that the extent of retention still depends on tissue disruption before pressing. Thus, the phenolic profile reported for cranberry pomace is shaped not only by genotype and analytical method, but also by the processing route through its effect on phase partitioning between juice and solid residue (Oszmiański et al. 2015).

Phenolic acids appear to represent a smaller fraction of the cranberry pomace phenolic pool than anthocyanins or PACs, but they are nevertheless consistently present. Oszmiański et al. (2015) identified several hydroxycinnamic acid derivatives in cranberry pomace from crushed fruits, including *p*‐coumaroyl‐hexose isomers, caffeoyl‐hexoside derivatives, sinapoyl‐hexose, chlorogenic acid, and caffeoyl‐dihexoside. Among these, *p*‐coumaroyl‐hexose isomer 1 (165.16 mg/100 g DM), caffeoyl‐hexoside isomer (74.59 mg/100 g DM), and sinapoyl‐hexose (56.54 mg/100 g DM) were among the most abundant components reported. In purified extract (PE) powders, Brzezowska et al. (2024) likewise quantified phenolic acids as a distinct chromatographic class, although without complete individual breakdown, supporting their continued presence after extraction and enrichment. Taken together, these findings indicate that phenolic acids are a regular but quantitatively secondary component of cranberry pomace phenolics, occurring mainly as substituted hydroxycinnamic acid derivatives rather than as one dominant free acid (Brzezowska et al. 2024; Oszmiański et al. 2015).

Anthocyanins constitute one of the most characteristic phenolic subclasses in cranberry pomace. Across studies, the anthocyanin profile is dominated by cyanidin‐ and peonidin‐based glycosides, with galactosides and arabinosides generally exceeding glucosides (Table 1). This pattern is evident in conventional PEs reported by White et al. (2010b), Kühn and Temelli (2017), and Ross et al. (2020). Considering values reported on a cranberry pomace DM basis, the principal anthocyanins are peonidin‐3‐*O*‐galactoside, cyanidin‐3‐*O*‐galactoside, cyanidin‐3‐*O*‐arabinoside, and peonidin‐3‐*O*‐arabinoside. In most studies, peonidin‐3‐*O*‐galactoside was reported at approximately 20–158 mg/100 g DM, cyanidin‐3‐*O*‐galactoside at 13–120 mg/100 g DM, cyanidin‐3‐*O*‐arabinoside at 45–85 mg/100 g DM, and peonidin‐3‐*O*‐arabinoside at 27–68 mg/100 g DM, whereas glucosides generally occurred at lower levels, such as 7–17 mg/100 g DM for peonidin‐3‐*O*‐glucoside and 4.5–7.4 mg/100 g DM for cyanidin‐3‐*O*‐glucoside. Notably, Oszmiański et al. (2015) reported much higher concentrations, reaching 4729.53 mg/100 g DM for peonidin‐3‐*O*‐galactoside and 3405.57 mg/100 g DM for cyanidin‐3‐*O*‐galactoside in pomace from crushed fruits. These values are markedly higher than those in most other studies. They may reflect differences in pomace generation and handling, including the use of laboratory pressing rather than industrial juice manufacture, the degree of tissue disruption before pressing, freeze‐drying of the pomace, and possible differences in the extent to which anthocyanin‐rich skins were retained in the residual fraction. Overall, cranberry pomace anthocyanins are compositionally defined by a recurrent pattern of cyanidin and peonidin conjugates with galactose and arabinose, whereas the absolute concentrations reported may vary substantially with processing history.

Tannins, particularly PACs, are another major phenolic feature of cranberry pomace and are especially important because cranberry is one of the distinctive dietary sources of A‐type procyanidins. Both compositional and depolymerization studies indicate that cranberry pomace contains a broad oligomeric distribution, including monomers, dimers, trimers, higher oligomers, and polymeric procyanidins (Table 1). A‐type dimers and trimers appear among the most prominent identifiable oligomeric species. For example, White et al. (2010b) reported A‐type dimers at 82.6 mg/100 g DM and A‐type trimers at 30.8 mg/100 g DM, together with smaller amounts of B‐type dimers and trimers, tetramers, pentamers, hexamers, and monomeric catechin/epicatechin. Likewise, White et al. (2010a) showed that after alkaline hydrolysis, procyanidin monomers, dimers, and trimers were recovered at 44.6–85.5, 248.9–519.3, and 85.6–144.5 mg/100 g DM, respectively, indicating that cranberry pomace contains not only extractable oligomers but also bound or poorly extractable procyanidins associated with the matrix. Thus, cranberry pomace tannins are compositionally defined by a structurally diverse procyanidin system extending from monomers to highly polymerized forms, with A‐type oligomers representing a characteristic component. Similar to anthocyanins, Oszmiański et al. (2015) reported substantially higher values than most other pomace studies, including procyanidin polymers at 2360 mg/100 g DM, A‐type trimers at 119.7 mg/100 g DM, A‐type dimers at 64.7 and 44.3 mg/100 g DM, B‐type dimers at 43.3 mg/100 g DM, (−)‐epicatechin at 73.6 mg/100 g DM, and (+)‐catechin at 28.98 mg/100 g DM. As in the anthocyanin fraction, these comparatively high values may reflect differences in pomace generation and handling, including laboratory‐scale pressing, the use of crushed rather than whole fruits, freeze‐drying of the pomace, and differential retention of skin‐ and seed‐associated phenolics in the residual fraction. Accordingly, the available evidence indicates that PACs are among the central phenolic classes in cranberry pomace, although their reported concentrations depend strongly on processing history and analytical approach.

Flavonols form another major non‐anthocyanin flavonoid subclass in cranberry pomace. The available evidence indicates a clear predominance of quercetin derivatives, accompanied by myricetin derivatives and smaller amounts of kaempferol derivatives (Table 1). In pomace DM data, total flavonols have generally been reported at approximately 2.86–3.08 mg quercetin equivalents (QE)/g DM. White et al. (2010b) identified a flavonol profile comprising quercetin, myricetin, quercetin‐3‐*O*‐benzoyl galactoside, quercetin‐3‐*O*‐rhamnoside, quercetin glycosides containing arabinose, galactose, and xylose, as well as several myricetin glycosides. Among these, quercetin (146.2 mg/100 g DM) and myricetin (55.6 mg/100 g DM) were the most abundant individually quantified flavonols, while quercetin glycosides such as quercetin‐3‐*O*‐benzoyl galactoside (27.5 mg/100 g DM), quercetin‐3‐*O*‐rhamnoside (18.5 mg/100 g DM), quercetin‐3‐*O*‐arabinofuranoside (16.7 mg/100 g DM), quercetin‐3‐*O*‐arabinopyranoside (15.2 mg/100 g DM), and quercetin‐3‐*O*‐galactoside (12.8 mg/100 g DM) were also prominent. These findings indicate that cranberry pomace flavonols are compositionally centered on quercetin conjugates, with myricetin derivatives making an important subsidiary contribution. Similar to anthocyanins and procyanidins, Oszmiański et al. (2015) reported substantially higher flavonol values than most other pomace studies. Their pomace from crushed fruits was dominated by myricetin‐3‐*O*‐pentoside derivatives, including isomer 1 at 1018.2 mg/100 g DM and isomer 2 at 628.6 mg/100 g DM, together with quercetin‐3‐*O*‐rhamnoside at 217.0 mg/100 g DM, methoxyquercetin‐hexoside at 97.3 mg/100 g DM, quercetin‐3‐*O*‐pentosides at 61.1–70.0 mg/100 g DM, and quercetin‐3‐*O*‐galactoside at 63.50 mg/100 g DM. Overall, although flavonols are not the quantitatively dominant phenolic class in cranberry pomace compared with anthocyanins or polymeric procyanidins, they represent a chemically diverse and recurrent fraction that contributes importantly to the overall phenolic profile.

Cranberry pomace shows a consistent phenolic profile dominated by cyanidin‐ and peonidin‐based anthocyanins, A‐type procyanidins and related flavan‐3‐ols, quercetin‐centered flavonols, and hydroxycinnamic acid derivatives. The most frequently reported constituents include peonidin‐3‐*O*‐galactoside, cyanidin‐3‐*O*‐galactoside, cyanidin‐3‐*O*‐arabinoside, peonidin‐3‐*O*‐arabinoside, A‐type procyanidin dimers and trimers, quercetin and its glycosides, myricetin derivatives, and *p*‐coumaroyl‐ and caffeoyl‐hexose derivatives. Within the broader group of pigmented berry pomaces (Table 2), cranberry pomace is therefore better regarded as a compositionally specialized matrix than as the richest source of total phenolics or anthocyanins. Its main distinction is the predominance of A‐type PACs, which separate it from raspberry pomace, characterized by ellagitannins; chokeberry pomace, rich in highly polymerized B‐type procyanidins; and blackcurrant, blueberry, and blackberry pomaces, where anthocyanin profiles are more central to compositional identity. Although absolute concentrations vary among studies, these recurring subclasses and marker compounds define the phenolic composition of cranberry pomace.

**TABLE 2:** Comparative overview of phenolic levels, major phenolic subclasses, and representative characteristic compounds in cranberry pomace and selected small‐berry pomaces.

Berry pomace	Reported phenolic level	Anthocyanin profile	Tannin and flavan‐3‐ol profile	Representative characteristic compounds	Selected references
Cranberry pomace	Reported total phenolics vary widely, from approximately 2–85 mg GAE/g DM, depending on pomace source, processing, extraction, and analytical approach	Contains cyanidin‐ and peonidin‐based glycosides; reported anthocyanin values commonly range from approximately 0.6–11 mg CGE/g DM	Mainly condensed tannins, especially A‐type proanthocyanidins; procyanidins with DP 1–6 have been reported, with A‐type dimers as major constituents	A‐type proanthocyanidin dimers and trimers; peonidin‐3‐*O*‐galactoside; cyanidin‐3‐*O*‐galactoside	Franco‐Londoño et al. (2025); Klavins et al. (2017); Ross et al. (2020); Saldaña et al. (2021); White et al. (2010b)
Raspberry pomace	Commonly approximately 15–40 mg GAE/g DM	Relatively low compared with total phenolics; reported values include approximately 0.27–1.88 mg CGE/g DM	Mainly hydrolysable tannins, especially lambertianin C and sanguiin H‐6; catechin, epicatechin, and procyanidin B1 occur as secondary constituents	Sanguiin H‐6; lambertianin C; ellagic acid derivatives	Ali Redha and Esatbeyoglu (2026)
Blueberry pomace	Reported total phenolics include approximately 5.46–13.4 mg/g DM, expressed as GAE or CE depending on the study	Contains delphinidin‐, cyanidin‐, petunidin‐, peonidin‐, and malvidin‐based glycosides; reported values include approximately 0.29–7.30 mg/g DM	Not typically tannin‐dominant; flavanols include catechin, epicatechin, procyanidin B1, and procyanidin B2	Chlorogenic acid; malvidin glycosides; delphinidin glycosides	Hu et al. (2026); Huang et al. (2025); Jaouhari et al. (2024); Lončarić et al. (2020)
Chokeberry pomace	Reported total phenolics approximately 22.9–63.0 mg GAE/g DM; chromatographic sums in dried fractions can be higher	Contains cyanidin‐3‐*O*‐galactoside and cyanidin‐3‐*O*‐arabinoside as major anthocyanins, with cyanidin‐3‐*O*‐glucoside and cyanidin‐3‐*O*‐xyloside also present	Mainly condensed tannins/procyanidins; epicatechin is a major structural unit and seedless fractions can contain highly polymerized proanthocyanidins	Cyanidin‐3‐*O*‐galactoside; cyanidin‐3‐*O*‐arabinoside; highly polymerized B‐type procyanidins	Saracila et al. (2024); Sidor and Gramza‐Michalowska (2019); Sójka et al. (2013)
Blackcurrant pomace	Reported total phenolics range from approximately 6.85–20 mg GAE/g DM; selected phenolic acids and flavonoids of approximately 1.02 mg/g have also been reported	Contains mainly delphinidin‐ and cyanidin‐based glycosides, especially delphinidin‐3‐*O*‐rutinoside, cyanidin‐3‐*O*‐rutinoside, delphinidin‐3‐*O*‐glucoside, and cyanidin‐3‐*O*‐glucoside	Condensed tannins/proanthocyanidins have been reported, with procyanidin/prodelphinidin composition; catechin, epicatechin, gallocatechin/epigallocatechin, and prodelphinidin‐type compounds are present	Delphinidin‐3‐*O*‐rutinoside; cyanidin‐3‐*O*‐rutinoside; delphinidin‐3‐*O*‐glucoside	dos Santos Lima et al. (2024); Guo et al. (2026); Untea et al. (2024)
Blackberry pomace	Reported total phenolics approximately 26.3–50.2 mg GAE/g DM	Contains cyanidin‐based anthocyanins; total monomeric anthocyanins of 8.43–17.31 mg CGE/g DM have been reported in extracts, while pomace powder values approximately 1.14–1.30 mg/g DM were reported in another study	Catechin is abundant, with reported values approximately 3.25–4.57 mg/g DM; epicatechin, rutin, myricetin, protocatechuic acid, and ellagic acid are also present	Cyanidin‐3‐*O*‐glucoside; ellagic acid; catechin	Čechovičienė et al. (2023); Jazić et al. (2018)

*Note*: Values are presented as general indicators rather than directly comparable absolute concentrations because cultivar, maturity, pressing conditions, drying method, particle size, extraction solvent, extraction conditions, analytical method, and expression basis can strongly influence reported values. For cranberry pomace, the broad phenolic range includes values reported for dried pomace materials extracted under different solvent and assisted‐extraction conditions, rather than a single standardized whole‐pomace value.

Abbreviations: CGE, cyanidin‐3‐*O*‐glucoside equivalents; DM, dry mass; DP, degree of polymerization; GAE, gallic acid equivalents.

### 2.2 Polysaccharides and Dietary Fiber

Polysaccharides and dietary fiber constitute a major proportion of cranberry pomace and contribute substantially to its compositional value. Because the pomace consists largely of skins, seeds, and residual cell‐wall material remaining after juice processing, it is particularly rich in structural carbohydrates. In dried cranberry pomace, insoluble fiber has been reported at 59.9–67.3 g/100 g and soluble fiber at 5.7–12.7 g/100 g, demonstrating that dietary fiber is a dominant compositional fraction and occurs predominantly in insoluble form (Jureviciute et al. 2022; White et al. 2010b; Bajerska et al. 2018).

The predominance of insoluble over soluble fiber is consistent with the origin of pomace as a cell wall‐rich residue. Nevertheless, the soluble fraction remains compositionally relevant because it forms part of the non‐starch polysaccharide system of the pomace and contributes to the overall heterogeneity of the carbohydrate matrix (Jureviciute et al. 2022; White et al. 2010b). In cranberry pomace, the water‐holding capacity was reported to be 2.78–4.24 g/g, the swelling capacity was 4.99–9.98 mL/g, and the oil‐binding capacity was 1.09–1.57 g/g. In comparison, the cholesterol‐binding capacity ranged from 21.11 to 23.13 mg/g (Jureviciute et al. 2022). These characteristics are consistent with a mixed fiber system containing both insoluble and soluble components.

Beyond total dietary fiber, cranberry pomace contains structurally complex cell wall polysaccharides, among which pectic polysaccharides appear to be particularly important. In cell wall material from Stevens cranberry, pectic polysaccharides accounted for more than 90% of the carbohydrates recovered in the hot buffer, chelating‐agent, and diluted‐alkali fractions (Spadoni Andreani et al. 2021a). Structural characterization further showed that the pectic fraction contained homogalacturonan with varying degrees of methyl esterification, together with branched rhamnogalacturonan‐I domains substituted with arabinan, galactan, and Type I arabinogalactan‐related side chains, with additional evidence suggesting arabinan structures bearing galactose‐containing branches (Spadoni Andreani, Karboune, and Liu 2021b). Thus, cranberry pomace is not simply a source of bulk fiber, but also a source of structurally diverse pectic polysaccharides.

The broader cell wall matrix of cranberry pomace also includes hemicellulosic polysaccharides, which contribute to the heterogeneity of the carbohydrate fraction. In the concentrated alkali fraction of Stevens cranberry cell wall material, glucomannan, xylan, and xyloglucan accounted for approximately 66% of the carbohydrates (Spadoni Andreani et al. 2021a). A broader comparative study further showed that pectic polysaccharides predominated in several cranberry pomace sources and their corresponding cell wall materials, whereas hemicellulosic polysaccharides constituted a substantial secondary fraction (Aleed et al. 2026). The same study also indicated that cranberry pomace contained both high‐molecular‐weight polysaccharides and lower molecular‐weight oligosaccharide fractions, further supporting the structural heterogeneity of its carbohydrate matrix.

These compositional features help explain the functional interest in cranberry pomace as a food ingredient. Enzymatic modification of cranberry pomace has been shown to alter the ratio of soluble to insoluble material, generate oligosaccharides, and improve water‐holding, oil‐holding, swelling, and emulsion‐stabilizing properties, while also enhancing the growth of selected probiotic bacteria (Jagelaviciute et al. 2022). Accordingly, the carbohydrate fraction of cranberry pomace may be viewed not only as a source of dietary fiber, but also as a reservoir of structurally and functionally diverse polysaccharides.

### 2.3 Lipids and Triterpenoid Constituents

Although the lipophilic fraction of cranberry pomace is smaller than its fiber‐rich and phenolic fractions, it remains compositionally important because it contains nutritionally and functionally relevant nonpolar constituents. This fraction is largely associated with the seed portion of the pomace and consists mainly of triacylglycerol‐rich oil characterized by a high degree of unsaturation. In cranberry seed oil, the predominant fatty acids were reported as linoleic acid (37%), α‐linolenic acid (31%), and oleic acid (24%), with smaller amounts of palmitic and stearic acids (Piasecka et al. 2023). A similar profile was reported for the lipophilic fraction recovered from cranberry pomace, in which linoleic acid accounted for 37%, linolenic acid for 32%, oleic acid for 22%, and palmitic acid for 4.3% (Tamkutė, Liepuoniūtė, et al. 2020). These findings indicate that the lipophilic fraction of cranberry pomace is dominated by unsaturated fatty acids, particularly polyunsaturated species.

Beyond the major fatty acids, the lipophilic fraction also contains a range of minor constituents of potential functional value. Fractionation studies have shown that cranberry pomace lipids contain not only triacylglycerols and fatty acids, but also minor lipophilic constituents such as carotenoids, tocopherols, phytosterols, squalene, and volatile compounds (Tamkutė, Pukalskas, et al. 2020). Supporting compositional data further showed total tocochromanols of 802.6 µg/100 g DM, including 491.1 µg/100 g DM tocopherols and 311.5 µg/100 g DM tocotrienols, with α‐tocopherol (463.5 µg/100 g DM) and γ‐tocotrienol (288.4 µg/100 g DM) as the dominant homologs (Kühn and Temelli 2017). Cranberry pomace therefore provides both bulk fibre and a structurally diverse range of pectic polysaccharides.

Cranberry pomace is also a good source of pentacyclic triterpenoids, which represent an additional class of lipophilic constituents distinct from glyceride‐based lipids. In a recent compositional study, the total triterpenoid content in cranberry pomace reached 64.1 mg/g DM, which was markedly higher than that in many cranberry fruit products (Xue et al. 2024). The major triterpenoids identified were ursolic acid, oleanolic acid, corosolic acid, and maslinic acid, together with esters of ursolic and oleanolic acids, with free ursolic acid being the major compound across the raw materials and products examined. In comparison, cranberry fruit samples contained 6.54–17.1 mg/g DM total triterpenoids, whereas whole berry products contained up to 2.67 mg/g DM, indicating marked enrichment of these constituents in pomace (Xue et al. 2024). Because triterpenoids are associated with the waxy peel layer of cranberry fruit, their high abundance in pomace is consistent with the retention of peel‐derived material during juice processing. Further characterization of the nonpolar fraction of cranberry pomace was recently reported by Mia (2026). Ultrasound‐assisted extraction (UAE) with acetone produced an enriched extract containing at least 51% triterpenoids and 4% phytosterols. On an extract‐mass basis, free ursolic acid was the predominant quantified triterpenoid at 263 mg/g, followed by hydroxycinnamoyl esters of ursolic and oleanolic acids at 148 mg/g and free oleanolic acid at 70 mg/g. The extract also contained neutral triterpenoids and related lipophilic constituents, including α‐ and β‐amyrin, lupeol, β‐sitosterol, campesterol, tocopherols, and tocotrienols. Although these values describe a selectively enriched extract rather than the original pomace, they demonstrate that the retained peel‐ and seed‐associated fraction comprises a broader and more concentrated triterpenoid profile than suggested by measurements of free triterpenoid acids alone.

## 3 Preprocessing and Pretreatment of Cranberry Pomace

### 3.1 Drying and Stabilization of Cranberry Pomace

Drying represents an important preprocessing step for cranberry pomace because of its high moisture content and susceptibility to quality deterioration during storage. Prior mechanical disruption may also influence subsequent stabilization. In American cranberry (*V. macrocarpon*), Oszmiański et al. (2015) showed that crushing fruits before pressing increased juice yield from 53.0 to 77.6% and reduced pomace yield from 47.0% to 22.4%. This treatment also affected the partitioning of constituents between juice and pomace, lowering the retention of sugars and organic acids in the pomace fraction to 7.3% and 18.2%, respectively, compared with 28.1% and 47.6% for pomace obtained from whole fruits. Nevertheless, total polyphenol retention in pomace remained high in both cases, reaching 78.9% after crushing and 95.4% after pressing whole fruits. The authors further noted that disrupted fruit tissues facilitated moisture removal, indicating that the extent of tissue breakdown before drying may influence both dehydration behavior and the compositional characteristics of the resulting pomace.

A more direct evaluation of drying conditions was later provided by Ross et al. (2020), who examined the stabilization of cranberry pomace by freeze‐drying and cabinet convection air drying. The cranberry pomace used in that study had an initial moisture content of 71.53% (wet basis), underscoring the need for rapid stabilization. Cabinet drying was performed at 50°C, 60°C, and 70°C under two loading densities, corresponding to 4.2 and 8.4 kg/m^2^ for half and full load, respectively, while freeze‐drying was used as a reference treatment. The drying time decreased markedly as the load density was reduced and the temperature increased. At half load, drying times declined from 420 min at 50°C to 240 min at 70°C, whereas full‐load samples required 1410 min under all three temperatures tested. This was accompanied by higher drying rates at half load (0.006–0.010 kg water/kg DM/min) than at full load (0.002 kg water/kg DM/min), indicating that bed thickness exerted a major influence on dehydration efficiency. Importantly, cabinet convection drying preserved much of the phenolic quality of cranberry pomace relative to freeze‐drying. Total phenolics in freeze‐dried pomace were reported as 15.17 mg GAE/g DM, while cabinet‐dried samples ranged from 13.54 to 14.97 mg GAE/g DM. Flavonol levels were likewise well maintained, varying from 2.86 to 3.03 mg QE/g DM, compared with 2.99 mg QE/g DM in freeze‐dried pomace. Anthocyanins were more sensitive but remained relatively stable under most conditions, declining from 1.47 mg CGE/g DM in freeze‐dried pomace to 1.07–1.20 mg CGE/g DM after cabinet drying, with a significant reduction becoming evident particularly at full load and 70°C. Antioxidant activity was also largely preserved, with 2,2′‐azino‐bis(3‐ethylbenzothiazoline‐6‐sulfonic acid) (ABTS) values of 88.85–93.01 µmol Trolox equivalents (TE)/g DM for cabinet‐dried samples compared with 96.10 µmol/g DM for freeze‐dried pomace and ferric reducing antioxidant power (FRAP) values of 67.13–70.81 µmol/g DM compared with 69.65 µmol/g DM for the freeze‐dried material. On this basis, cabinet air drying at 70°C and half load density appears to offer an efficient means of reducing drying time while maintaining phenolics and antioxidant activity at levels generally comparable to those of freeze‐dried pomace, thus providing a practical alternative for cranberry pomace stabilization.

Additional evidence from cranberry PE powders indicates that the drying method and temperature may also influence the retention of phenolic subclasses in downstream products. In powders obtained from PEs, freeze‐drying and vacuum drying at 60°C and 90°C yielded broadly comparable flavonol and antioxidant values, whereas vacuum drying at 90°C tended to reduce anthocyanins and phenolic acids more markedly, particularly in acidified ethanol extracts (Brzezowska et al. 2024). Thus, while higher‐temperature vacuum drying may improve some physical properties of powders, milder conditions appear more favorable for preserving thermolabile phenolic constituents in pomace‐derived extracts.

### 3.2 Solid‐State Fermentation as a Bioprocessing Pretreatment

Solid‐state fermentation (SSF) has been explored in a limited number of early studies as a bioprocessing pretreatment for cranberry pomace. The rationale behind this approach was that fungal enzymes, particularly β‐glucosidase, could hydrolyze phenolic glycosides and thereby mobilize free phenolic aglycones and related antioxidant constituents. Zheng and Shetty (2000) examined the SSF of cranberry pomace with the food‐grade fungus *Lentinus edodes* and reported that total free phenolics reached a maximum of 0.5 mg/g pomace after 50 days of cultivation, while β‐glucosidase activity increased to approximately 9 U/g pomace. The major free phenolic acids identified in the fermented extract were gallic, chlorogenic, *p*‐hydroxybenzoic, and *p*‐coumaric acids, supporting the view that enzymatic hydrolysis contributed to the release of soluble phenolics from the pomace matrix.

Subsequent work by Vattem and Shetty (2003) extended this concept by relating SSF‐mediated phenolic mobilization to antioxidant properties. Using *L. edodes*, these authors reported increases in extractable phenolics and antioxidant capacity during the course of solid‐state growth, with β‐glucosidase activity increasing by 78‐ to 110‐fold depending on the nitrogen treatment. In the same study, cranberry pomace was enriched with ellagic acid to as much as 350 µg/g DM pomace, suggesting that SSF may also promote the release of diphenyl compounds, possibly through hydrolysis of ellagitannins and related esters. A related study employing *Rhizopus oligosporus* further confirmed the potential of SSF as a value‐addition strategy (Vattem and Shetty 2002). Solid‐state growth for 16 days increased water‐extractable phenolics by 15%–26% by Day 10, while β‐glucosidase activity rose by 60‐fold with NH_4_NO_3_ and by more than 100‐fold with fish protein hydrolysate (initially used as a supplemental nitrogen source in the SSF medium). Ellagic acid enrichment was again observed, reaching 375 µg/g DM pomace, together with an improved antioxidant protection factor. Thus, these studies indicate that SSF can mobilize phenolic aglycones and enrich ellagic acid in cranberry pomace, but its long processing time (16–50 days) limits practical relevance compared with direct drying and solvent extraction.

## 4 Extraction of Valuable Constituents From Cranberry Pomace

The cranberry extraction method should be selected according to the target fraction. Phenolics are commonly recovered using hydroalcoholic, acidified, ultrasound‐assisted, or pressurized solvent systems; seed lipids by supercritical CO_2_; and pectic or cell wall polysaccharides by hot‐water extraction, acid extraction, alkaline extraction, microwave‐assisted extraction (MAE), or enzyme‐assisted extraction. Table 3 summarizes these approaches, target fractions, and their food‐related advantages and limitations.

**TABLE 3:** Extraction and recovery approaches applied to cranberry pomace and their relevance for food‐oriented valorization.

Extraction method	Target fraction	Advantages	Limitations and considerations
Conventional hydroalcoholic extraction	Broad phenolic‐rich extracts containing anthocyanins, flavonols, phenolic acids, and some proanthocyanidins	Simple, scalable, and food‐relevant when ethanol–water is used; good baseline method for cranberry pomace phenolics	Limited selectivity; co‐extracts sugars, organic acids, and mixed phenolics; may not efficiently recover matrix‐bound procyanidins
Acidified hydroalcoholic extraction	Anthocyanin‐rich extracts and pigment‐retaining phenolic extracts	Acidic conditions help stabilize cranberry anthocyanins, especially cyanidin and peonidin glycosides	Conditions suitable for anthocyanins are not always optimal for proanthocyanidins; strong acidification may reduce clean‐label appeal
Ultrasound‐assisted extraction	Total phenolics, anthocyanins, and proanthocyanidins from cranberry pomace	Shortens extraction time and improves release from the skin–seed matrix; compatible with ethanol–water systems	Temperature must be controlled because cranberry anthocyanins are heat‐sensitive; excessive sonication may promote degradation
High‐intensity ultrasound with NADES	Green extraction of anthocyanins and procyanidins	Promising alternative to conventional organic solvents; relevant for greener cranberry pomace valorization	NADES viscosity, pH, solvent recovery, extract standardization, and food‐regulatory acceptance remain challenges
Pressurized liquid/fluid extraction	Anthocyanins, total phenolics, and phenolic‐rich extracts	Improves extraction efficiency and allows tuning of solvent composition and temperature; pressurized ethanol is useful for anthocyanins	Different cranberry phenolic targets require different optima; high temperature may affect thermolabile anthocyanins
Supercritical CO_2_ extraction	Seed‐associated lipophilic fraction, including cranberry seed oil, unsaturated fatty acids, tocochromanols, carotenoids, phytosterols, and squalene	Highly relevant because cranberry pomace contains seeds; useful for recovering oil before further valorization of the remaining matrix	Not suitable alone for polar polyphenols such as anthocyanins and proanthocyanidins
Sequential SC‐CO_2_ + pressurized ethanol/water extraction	Stepwise recovery of lipids followed by phenolic‐rich and hydrophilic fractions	Strong fit for cranberry pomace because it uses the same biomass stream for multiple fractions	More complex and costly than single‐step ethanol extraction; less practical for simple food‐ingredient production
Microwave‐assisted extraction	Pectin, pectic oligosaccharides, cell‐wall polysaccharides, and mixed polyphenol–polysaccharide fractions	Highly relevant because cranberry pomace is fiber‐ and pectin‐rich; can reduce time and solvent/energy use	Mainly suited for carbohydrate‐rich fractions rather than selective phenolic extraction; conditions can modify pectin structure
Enzyme‐assisted extraction	Pectic oligosaccharides, soluble fiber fractions, and modified pomace ingredients	Food‐science relevant for producing functional fiber/prebiotic‐type ingredients with improved hydration and emulsifying properties	Enzyme cost, specificity, incubation time, and process control may limit industrial simplicity
Acid extraction	Pectin and pectic polysaccharides from cranberry pomace cell walls	Directly relevant to edible coatings, films, and food stabilizer applications	Acid conditions affect molecular weight, degree of esterification, and final functionality of cranberry pomace pectin
Alkaline extraction	Matrix‐associated procyanidins and cell‐wall polysaccharide fractions	Useful for releasing cranberry procyanidins bound or tightly associated with the pomace matrix	Harsh and less food‐friendly; may alter native phenolic structures, so it is better for analytical or mechanistic studies than clean‐label ingredients
Hot‐water extraction	Water‐soluble polysaccharides, oligosaccharides, and some hydrophilic phenolics	Simple, food‐compatible, and clean‐label; suitable for mild recovery of soluble fiber‐type fractions	Lower efficiency for many cranberry phenolics, especially proanthocyanidins, flavonols, and anthocyanin‐rich extracts

### 4.1 Extraction of Polyphenols From Cranberry Pomace

#### 4.1.1 Extraction of Total Phenolics and Phenolic‐Rich Extracts

Recovery of total phenolics from cranberry pomace has generally been directed toward the production of broad‐spectrum phenolic‐rich extracts rather than highly selective isolates. This is attributable to the compositional complexity of the matrix, in which phenolic acids, flavonols, anthocyanins, and flavan‐3‐ol‐derived compounds may coexist and respond differently to solvent polarity, acidification, and extraction intensity. Accordingly, the current studies have mainly examined how conventional solvent systems and subsequent process intensification influence overall phenolic recovery, antioxidant capacity, and extract quality.

Among the more conventional approaches, ethanol‐based solid‒liquid extraction has been used to obtain phenolic‐rich fractions from cranberry press cake. In a pilot‐scale study, cranberry press cake was extracted with 95% ethanol either by three successive soaking and decanting steps or by a single soaking step followed by decanting and bladder pressing (Harrison et al. 2013). Total dry extract recovery reached 87.8 g/kg press cake in the first process and 55.8 g/kg press cake in the second, while the corresponding values based on fresh cranberries were 80.7 and 72.2 g/kg, respectively. The resulting press cake extracts contained approximately three times the phenolic acids, tartaric esters, and antioxidant activity of the corresponding juice powders, together with approximately five times the flavonols and sixfold to tenfold higher anthocyanin levels. Tannins represented a substantial proportion of the extractable phenolic material, reaching 88–108 mg catechin equivalents (CE)/g in selected extracts and accounting for 68%–72% of the measured phenolic acids. These findings indicate that ethanol extraction can effectively concentrate a broad range of phenolic constituents from cranberry processing residues, although extract composition remains influenced by the extraction sequence and pressing strategy.

A solvent‐based approach was also applied to American cranberry pomace using 30% acetone, 50% ethanol, and acidified 50% ethanol (pH 2), followed by XAD‐16 resin treatment and drying (Brzezowska et al. 2024). The sum of phenolics in the resulting powders was generally in the range of 10.1–11.0 g/100 g DM. Acidified 50% ethanol improved the recovery of flavan‐3‐ols, while 50% ethanol and its acidified counterpart produced, on average, approximately 23% higher anthocyanin content than 30% acetone. These findings indicate that solvent composition and acidification markedly influence the distribution of phenolic subclasses recovered from cranberry pomace.

Process intensification has been investigated to improve extraction efficiency and reduce time and solvent consumption. For American cranberry press residues, comparison of UAE, MAE, and Soxhlet extraction showed that UAE had the highest practical potential because of its rapidity, convenience, and comparatively low cost (Klavins et al. 2017). In that study, aqueous ethanol and methanol in the presence of acid were identified as the most suitable solvents, with 1% HCl preferred for non‐anthocyanin polyphenols and 1% trifluoroacetic acid (TFA) for anthocyanins. Ultrasound treatment for approximately 15–25 min at a solid‐to‐liquid ratio of approximately 1:90 to 1:120 gave the highest extraction yields. These observations support the view that assisted extraction can enhance the recovery of broad phenolic fractions from cranberry pomace while allowing better control over extraction severity and solvent use than prolonged conventional techniques.

More advanced high‐pressure and integrated recovery strategies have further extended the extraction options for cranberry pomace. In American cranberry (*V. macrocarpon*), consecutive extraction using supercritical carbon dioxide (SC‐CO_2_) for lipids followed by ternary compressed fluids composed of CO_2_, ethanol, and water enabled recovery of phenolic‐rich extracts with total phenolic contents, anthocyanin concentrations, and antioxidant activities similar to those obtained by conventional extraction with 70% acidified aqueous ethanol, while substantially reducing ethanol consumption (Kühn and Temelli 2017). The inclusion of water in the compressed fluid system improved phenolic recovery and antioxidant activity even at high CO_2_ proportions, whereas CO_2_ with water favored the highest anthocyanin concentrations. The study therefore demonstrated that compressed fluid extraction may provide a more environmentally attractive route for broad phenolic recovery from cranberry pomace without marked loss of extract quality.

A related biorefining concept was proposed by Tamkutė, Liepuoniūtė, et al. (2020), in which cranberry pomace was first defatted by supercritical fluid extraction and then subjected sequentially to pressurized liquid extraction (PLE) with ethanol and water. Under optimized conditions, PLE‐EtOH at 83°C for 3 × 15 min recovered 55.89% of soluble material, while subsequent PLE‐H_2_O at 130°C for 3 × 10 min yielded an additional 6.5% extract from the residue. Although the aqueous fraction exhibited the strongest antioxidant capacity, the ethanolic PLE fraction recovered the majority of the polyphenolics and was also effective for anthocyanins and PACs. This sequential approach is noteworthy because it integrates broad phenolic recovery into a zero‐waste valorization scheme in which lipophilic and hydrophilic fractions are obtained consecutively from the same pomace stream.

Recovery of total phenolics and phenolic‐rich extracts from cranberry pomace has progressed from conventional ethanol‐based extraction toward assisted and high‐pressure approaches that improve extraction efficiency and, in some cases, reduce organic solvent consumption. Conventional solvent systems remain effective for obtaining broad phenolic concentrates, whereas UAE, compressed fluids, and PLE provide greater flexibility in modulating selectivity, extraction time, and process sustainability.

#### 4.1.2 Extraction of Anthocyanins

Anthocyanins constitute an important subgroup of cranberry pomace phenolics and differ from broader phenolic fractions in that their recovery requires greater attention to solvent acidification, polarity, and process severity. Because these pigments are water soluble yet chemically labile, extraction conditions that are suitable for total phenolics do not necessarily maximize anthocyanin yield.

Among the more conventional approaches, anthocyanin‐rich fractions have been obtained using acidified hydroalcoholic systems. An early study on *V. macrocarpon* prepared separate water‐soluble, apolar phenolic, and anthocyanin‐rich extracts from fruit and pomace, with the anthocyanin‐rich fraction recovered using methanol/water/acetic acid (85/14.5/0.5, v/v/v) (Caillet, Lorenzo, Côté, Sylvain, et al. 2012). This study illustrates the suitability of acidified hydroalcoholic solvents for obtaining anthocyanin‐enriched cranberry PEs.

Optimization of solvent composition for anthocyanin recovery was later examined in berry press residues by response surface methodology (RSM). In *V. macrocarpon* and *V. oxycoccos*, the extraction conditions favoring anthocyanins were shown to differ from those favoring total polyphenols, confirming the need for target‐specific optimization (Klavins et al. 2018). Higher solvent concentrations generally favored anthocyanin recovery, with 40%–70% ethanol or 40%–60% methanol in the presence of acid giving the best results for anthocyanin extraction. Under the optimized conditions, the anthocyanin content in press residue extracts reached 43.53 mg/g in cranberry and 7.08 mg/g in American cranberry, and the anthocyanin profile remained comparable between berries and their press residues, suggesting that these pigments were largely retained during juice processing and drying.

More advanced approaches have relied on pressurized fluids to improve anthocyanin recovery from cranberry pomace. Pressurized fluid extraction (PFE) with water, 30%–100% ethanol, or 5% citric acid showed that 100% ethanol was the most effective solvent for anthocyanin recovery, yielding 6.02–8.42 mg CGE/g DM at 40°C–120°C and 50 bar, with temperature exerting little effect within that range (Saldaña et al. 2021). In contrast, maximum total phenolics were obtained under different conditions, namely, 30% ethanol at 140°C and 50 bar, again illustrating that anthocyanin extraction is not optimized by the same conditions as broad phenolic recovery. Irrespective of treatment, the major anthocyanins recovered were cyanidin‐3‐*O*‐galactoside, cyanidin‐3‐*O*‐arabinoside, peonidin‐3‐*O*‐galactoside, and peonidin‐3‐*O*‐arabinoside. These findings indicate that pressurized ethanol provides a useful means of selectively recovering anthocyanins from cranberry pomace while preserving the characteristic anthocyanin profile.

A more recent direction has been the use of green solvents and ultrasound assistance. In cranberry pomace (*V. macrocarpon*), high‐intensity ultrasound (HIUS) combined with natural deep eutectic solvents (NADES) was evaluated using several choline chloride‐based formulations (Franco‐Londoño et al. 2025). The highest total anthocyanin content obtained during HIUS extraction was 0.86 mg CGE/g sample with ethanol, while the choline chloride + glycerol mixture yielded a comparable value of 0.78 mg CGE/g sample. The same study showed that NADES performance depended strongly on physicochemical properties such as pH and viscosity, with mixtures in the pH range of 2.0–4.0 and viscosities below 20 mPa s giving more favorable anthocyanin recovery. In PLE with NADES, choline chloride + glycerol also outperformed choline chloride + acetic acid, reaching 0.63 mg CGE/g after 20 min, compared with only 0.13 mg CGE/g for the latter system. These results suggest that NADES‐based extraction, particularly when coupled with HIUS, may provide a promising green alternative for anthocyanin recovery, although the yields remained comparable rather than clearly exceeding those obtained with ethanol.

In general, extraction of anthocyanins from cranberry pomace should be approached under conditions distinct from those used for broad phenolic extraction, with particular attention to solvent acidification, ethanol concentration, and process severity. When selective anthocyanin recovery is the main objective, pressurized ethanol and HIUS‐assisted NADES systems appear to be promising options. However, conventional acidified hydroalcoholic solvents remain suitable where simpler extraction schemes are preferred and highly selective recovery is not needed.

#### 4.1.3 Extraction of Tannins and PACs

Tannins, particularly PACs, constitute one of the most distinctive phenolic fractions in cranberry pomace and are of special interest because of their structural diversity, degree of polymerization (DP), and well‐recognized biofunctional relevance. In cranberry, these compounds are notable not only for their abundance in pomace but also for the occurrence of A‐type PACs, which have attracted considerable attention in relation to functional and health‐promoting properties. From an extraction standpoint, their recovery is more complex than that of total phenolics or anthocyanins because PACs may occur in both extractable and matrix‐associated forms, and selective enrichment is often required when structurally defined fractions are desired.

An early study demonstrated that a portion of cranberry pomace procyanidins may remain in a bound form and therefore escape recovery by conventional solvent extraction alone (White et al. 2010c). Using cranberry (*V. macrocarpon*) pomace, alkaline hydrolysis with 2 N NaOH at 60°C for 15 min, followed by acidification and ethyl acetate extraction, released low‐molecular‐weight procyanidins that were not recovered efficiently by the conventional acetone/water/acetic acid (70:29.5:0.5, v/v/v) system. Relative to the conventional extract, alkaline treatment increased the recovery of procyanidins from DP1 to DP6 by 9.4‐fold, with the greatest increases observed for DP1 monomers (14.9‐fold) and A‐type DP2 dimers (8.4‐fold). Even when polymeric fractions were included, total procyanidins increased from 12.92 to 16.85 mg/g DM, and further treatment of the residue remaining after conventional extraction released 7.16 mg/g DM of additional DP1–DP6 procyanidins compared with only 1.66 mg/g DM recovered by the initial conventional method. These findings indicate that part of the cranberry pomace procyanidin fraction is tightly associated with the cell wall matrix and may require chemical disruption for effective release.

More recent work has focused on targeted extraction optimization for soluble cranberry procyanidins (Klavins et al. 2022). In American cranberry press residues, RSM showed that procyanidin recovery was maximized with an extraction solvent containing approximately 53% acetone, whereas the addition of acidifying agents did not significantly improve extraction yield. This is noteworthy because it differs from the behavior observed for broader polyphenolic extraction, where acidification may enhance recovery. The optimized extract was subsequently used for purification and detailed characterization, and the resulting procyanidin‐rich fractions contained 78 individual procyanidins, of which 65 were A‐type, with a DP of up to 9. These results show that cranberry press residues can serve as an important source of structurally diverse procyanidins and that solvent optimization alone is insufficient when the objective is to obtain highly enriched tannin fractions.

Purification has therefore become a central part of cranberry procyanidin recovery. A particularly important contribution was provided by Gao et al. (2018), who coupled ultrasound‐assisted water extraction with macroporous resin adsorption/desorption and compared five Amberlite resins, namely, XAD‐7HP, XAD‐761, XAD‐16N, XAD‐1180, and FPX‐66. Among these materials, XAD‐7HP showed the highest adsorption capacity for both procyanidins (52.2 mg/g resin) and total phenolics (99.1 mg/g resin), whereas XAD‐761 showed the lowest performance. The superior behavior of XAD‐7HP was attributed to its favorable physicochemical properties, and the adsorption of procyanidins on this resin followed pseudo‐second‐order kinetics, while the equilibrium data were better described by the Langmuir isotherm, suggesting monolayer adsorption on a relatively homogeneous surface. Under dynamic conditions, adsorption and desorption flow rates of 7 and 8 mL/min, respectively, were selected as optimal, and complete desorption was achieved within 9 bed volumes using 95% ethanol acidified with 1% formic acid. Resin treatment increased total procyanidins by 5.56‐fold based on high‐performance liquid chromatography (HPLC) and 4.57‐fold based on 4‐dimethylaminocinnamaldehyde (DMAC) analysis, while total phenolics increased 4.73‐fold. In addition, flavonols and phenolic acids increased by twofold to sevenfold, whereas sugars such as fructose, glucose, and sucrose were no longer detected after purification. Importantly, polymers with DPs > 10 accounted for 77.6% of the total procyanidins in the concentrated extract, indicating strong enrichment of higher‐molecular‐weight procyanidins. Thus, this study demonstrated not only that resin purification can markedly enhance procyanidin concentration, but also that resin choice strongly affects separation efficiency and the quality of the resulting tannin‐rich fraction.

A more elaborate purification sequence was later applied by Klavins et al. (2022), beginning with XAD7‐HP to remove carbohydrates and organic acids, followed by Sephadex LH‐20 (LH‐20) and finally silica gel fractionation to obtain procyanidin‐rich fractions of improved compositional definition. This multistep procedure confirmed that purification is not merely a supplementary operation for cranberry tannins but an essential step when the objective is to isolate fractions enriched in procyanidins of different degrees of polymerization and suitable for advanced structural characterization. At the same time, the study also pointed out a practical limitation of XAD7‐HP in this context, namely, partial loss of target procyanidins during washing, attributed to the comparatively large resin particle size and lower binding capacity relative to finer adsorbents.

PAC recovery from cranberry pomace is best achieved by selective extraction followed by purification. For the readily extractable fraction, approximately 50% aqueous acetone appears preferable to strongly acidified systems, as it maximized the procyanidin yield without additional benefit from acidification (Klavins et al. 2022). However, a portion of cranberry procyanidins remains associated with the pomace matrix and may require alkaline hydrolysis or other disruptive treatments for effective release, particularly when bound low‐molecular‐weight oligomers are of interest (White et al. 2010c). When the objective is to obtain highly enriched tannin fractions rather than crude phenolic extracts, purification becomes essential. Among the resins evaluated, Amberlite XAD‐7HP showed the most favorable adsorption and desorption performance for cranberry procyanidins and therefore appears to be a suitable first purification step (Gao et al. 2018). Further fractionation with adsorbents such as LH‐20 and silica gel may then be employed when procyanidin‐rich fractions of improved compositional definition and DP selectivity are required (Klavins et al. 2022). Thus, the most effective strategy seems to depend on the intended end use. Moderate‐strength acetone‐based extraction may be sufficient for the recovery of crude procyanidin‐rich extracts, whereas structurally defined or highly concentrated fractions require sequential purification and fractionation.

### 4.2 Recovery of Pectin, Pectic Oligosaccharides, and Cell Wall Polysaccharides From Cranberry Pomace

Cranberry pomace is a cell wall‐rich by‐product in which structural carbohydrates represent a major fraction of the dry material. Within this carbohydrate matrix, pectin refers principally to galacturonic acid‐rich cell wall polysaccharides that may include homogalacturonan and rhamnogalacturonan domains, whereas pectic oligosaccharides are lower molecular‐weight fragments generated from partial depolymerization of these polymers. The broader term cell wall polysaccharides includes not only pectic substances but also hemicellulosic components such as xylan, xyloglucan, and glucomannan. These fractions are of interest because they may serve as dietary fiber ingredients, prebiotic substrates, gelling or stabilizing agents, and value‐added food biopolymers. In cranberry pomace, cell wall polysaccharides have been reported to constitute most of the pomace solids, with pectic polysaccharides representing a major fraction of the extractable carbohydrate material.

Pectin is one of the principal polysaccharide targets in cranberry pomace. In Canadian *V. macrocarpon*, microwave‐intensified extraction improved pectin recovery by at least 17%, reduced processing time by approximately 90%, lowered solvent use by approximately 27%, and decreased energy consumption by approximately 48% relative to conventional heating (Adetunji 2016). The recovered pectin contained 43%–52% galacturonic acid, had a viscosity‐average molecular weight of approximately 39 kDa, and was described as a low‐methoxyl polysaccharide with potential viscosity‐enhancing functionality. A more recent study further expanded the recovery strategies by comparing hot water, oxalic acid, and choline chloride/oxalic acid‐based NADES systems, with or without ultrasound assistance, followed by dialysis purification (F. Wang et al. 2025). In that work, the pectin yield ranged from 4.85% for water extraction to 27.81% for oxalic acid extraction, while NADES extraction gave a 15.56% yield, increasing to 17.32% when combined with ultrasound. The galacturonic acid content varied from 60.33% for water‐extracted pectin to 81.90% for oxalic acid‐extracted material, whereas NADES‐extracted samples showed intermediate values of 74.9%–78.5%. In addition, the degree of esterification ranged from 34.34% to 67.47%, indicating that the extraction medium affected not only the pectin yield, but also the structural characteristics of the isolated fraction.

Recovery of cranberry pomace polysaccharides has also been directed toward pectic oligosaccharides and mixed oligo/polysaccharide fractions. Microwave‐assisted alkaline extraction and enzymatic treatments applied to American cranberry pomace yielded carbohydrate fractions that differed not only in amount but also in molecular profile and composition (Spadoni Andreani and Karboune 2020). The MAE method produced, on average, 21.3% total sugars and generated extracts composed largely of oligosaccharides with a DP of 7–10, whereas multienzyme systems yielded 23.4%–42.0% total sugars but mainly shorter oligosaccharides with a DP of 2–5. The microwave‐assisted extracts were enriched in pectic oligosaccharides, while enzymatic extracts were relatively richer in glucans and contained lower proportions of rhamnose and galactose, indicating that the extraction strategy strongly influenced the type of carbohydrate fraction recovered.

A related example is provided by the MAE of mixed pectic oligo/polysaccharide fractions together with polyphenols from *V. macrocarpon* pomace (Davis et al. 2021). In that study, acid extraction gave the lowest yields (3.4%–4.9%, w/w), whereas alkaline extraction at lower microwave power yielded 12.6%. Under optimized sequential acidic/alkaline conditions, the extract yield reached 28.65%, with polyphenol and oligo/polysaccharide recoveries of 57.0% and 10.6%, respectively. The carbohydrate portion of the optimized extract was described as an arabinan‐rich rhamnogalacturonan I‐type pectic fraction, showing that cranberry pomace may serve as a source of structurally defined branched pectic materials rather than only crude pectin.

Broader cell wall polysaccharide recovery has likewise been investigated through sequential fractionation strategies. Using pomace from Stevens cranberry (*V. macrocarpon* var. Stevens), Spadoni Andreani et al. (2021b) showed that different extractants recovered distinct cell wall populations. Pectic polysaccharides represented more than 90% of the carbohydrates in the hot buffer, chelating agent, and diluted alkali extracts, whereas the concentrated alkali fraction was enriched in hemicellulosic components, with glucomannan, xylan, and xyloglucan accounting for approximately 66% of its carbohydrates. The chelating agent extract gave the highest yield (11.0%, w/w), and the total yield of extracted polysaccharides reached 21.8% of alcohol‐insoluble solids (AISs) DM. High‐molecular‐weight polysaccharides were identified in all fractions, confirming that cranberry pomace contains multiple structurally distinct cell wall populations that can be selectively recovered by stepwise extraction.

A broader comparative perspective was provided in a proof article by Aleed et al. (2026), in which several cranberry pomace sources and extraction approaches were evaluated, including hot water, alkaline, sequential acid/alkaline, and enzymatic treatments. Across these materials, pectic polysaccharides predominated in the pomaces and their corresponding cell wall materials, and the extraction method had a marked effect on the type of fraction recovered. Sequential (55.3%) and enzymatic (49.2%) approaches yielded the highest proportions of pectic polysaccharides, whereas water (62.8%) and alkaline (66.5%) extracts were relatively richer in hemicellulosic polysaccharides. High‐molecular‐weight fractions in the 100–500 kDa and 10–100 kDa ranges were abundant, particularly in alkaline extracts, while oligosaccharides of 0.5–3 kDa were produced predominantly by sequential, water, and enzymatic methods. These findings further emphasize that cranberry pomace polysaccharide recovery is highly method‐dependent and that extraction conditions govern both the relative abundance of pectic versus hemicellulosic fractions and the molecular weight distribution of the recovered materials.

Overall, cranberry pomace is a promising source of pectin, pectic oligosaccharides, and structurally diverse cell wall polysaccharides. Recovery approaches range from direct pectin extraction to assisted, sequential, and enzymatic strategies capable of generating fractions with more defined composition and molecular size. In general, acidified and chelating conditions favor the recovery of pectic materials, alkaline treatments facilitate the release of broader cell wall polysaccharides but may also modify their structure, and sequential or enzymatic approaches appear particularly useful when the objective is to obtain oligosaccharide‐rich or highly branched pectic fractions for value‐added applications.

### 4.3 Recovery and Fractionation of Lipophilic Constituents From Cranberry Pomace

Although cranberry pomace is commonly discussed as a source of phenolics and dietary fiber, it also contains a valuable lipophilic fraction, largely associated with the seed portion of the pomace. This fraction consists mainly of triacylglycerols rich in unsaturated fatty acids, together with minor bioactive constituents such as tocopherols, tocotrienols, carotenoids, phytosterols, squalene, and volatile compounds (Kühn and Temelli 2017; Piasecka et al. 2023; Tamkutė, Pukalskas, et al. 2020). Accordingly, the recovery of lipophilic constituents from cranberry pomace has been pursued both for the isolation of crude oil‐rich extracts and for subsequent fractionation into streams with different compositional and functional characteristics.

SC‐CO_2_ extraction has been the principal approach used for recovering lipophilic constituents from cranberry pomace. In American cranberry, SC‐CO_2_ extraction yielded a pomace fraction containing 9.00% lipids on a DM basis, with linoleic, linolenic, and oleic acids as the dominant fatty acids and total tocochromanols reaching 802.6 µg/100 g DM (Kühn and Temelli 2017). Within this fraction, linoleic acid accounted for 40.03% of the total fatty acids, followed by linolenic acid at 32.11% and oleic acid at 17.37%, while the n‐6/n‐3 ratio was reported as 1.3:1, indicating a nutritionally favorable profile. In a later consecutive biorefining study, SC‐CO_2_ extraction of cranberry pomace gave the highest lipophilic yield of 11.10 ± 0.15% at 42.4 MPa, 53°C, and 158 min, and the extracted oil was likewise dominated by linoleic (36.58%), linolenic (32.44%), oleic (21.79%), and palmitic (4.36%) acids (Tamkutė, Liepuoniūtė, et al. 2020). Polyunsaturated fatty acids represented 69.58 g/100 g, whereas saturated fatty acids accounted for only 6.44 g/100 g, and the n‐6/n‐3 ratio was approximately 1.1:1, further emphasizing the nutritional value of the recovered oil.

Recovery of cranberry pomace lipids has also been followed by fractionation to obtain extracts enriched in selected lipophilic constituents. Using the same optimized SC‐CO_2_ conditions (42.4 MPa, 53°C), online separation at 7 MPa with first‐separator temperatures ranging from 0°C to −30°C enabled the production of heavier and lighter fractions with distinct compositions (Tamkutė, Pukalskas, et al. 2020). Depending on the separation temperature, 61%–74% of the extract precipitated in the first separator during pure SC‐CO_2_ extraction. The addition of 5% ethanol as a cosolvent increased the extract yield, and at lower separator temperatures, the heavier fraction became 2.9‐ to 3.5‐fold greater than the lighter fraction. The lighter fraction showed stronger antioxidant capacity, whereas the heavier fraction was richer in carotenoids and, under some conditions, showed markedly higher squalene concentration, reaching up to 12‐fold enrichment. Differences in triacylglycerol and fatty acid composition between fractions were less pronounced, although heavier fractions tended to contain more saturated triacylglycerols, while lighter fractions were relatively richer in polyunsaturated species. This study demonstrates that cranberry pomace lipids may be recovered not only as a crude oil‐rich fraction, but also as more specialized lipophilic streams differing in fatty acid distribution and minor bioactive composition.

A more focused lipophilic stream is represented by cranberry seed oil because the seeds are the principal repositories of lipids within pomace. The UAE of oil from cranberry seeds gave an optimized predicted yield of 22.55 ± 0.36%, with an actual yield of 21.98 ± 0.08%, at 95% amplitude and 11.38 min extraction time (Piasecka et al. 2023). The recovered oil from American cranberry seeds was characterized by high proportions of linoleic (36.78%–36.81%), α‐linolenic (30.68%–31.33%), and oleic acids (24.08%–24.44%), together with smaller amounts of palmitic and stearic acids. Sonication also influenced some quality‐related attributes, including oxidation behavior, fatty acid distribution in triacylglycerols, and melting characteristics, while scanning electron microscopy showed pore enlargement and disruption of fat agglomerates in the seed matrix. These results indicate that cranberry seeds, whether considered separately or as part of pomace, constitute an important source of nutritionally valuable oil and that physical assistance such as ultrasound may improve oil recovery from this lipophilic fraction.

### 4.4 Emerging Extraction Opportunities and Industrial Translation

Several conventional and intensified extraction methods have been investigated for cranberry pomace, whereas technologies tested in other berry pomaces remain unexplored. These studies suggest future directions, but cranberry‐specific validation is needed, particularly for anthocyanin and A‐type PAC recovery and stability.

Pulsed electric field (PEF) treatment appears particularly promising as a pretreatment to enhance mass transfer before food‐grade solid–liquid extraction. In blueberry processing, PEF increased juice yield and improved subsequent recovery of phenolics and anthocyanins from press cake (Bobinaitė et al. 2015). In fresh grape pomace, treatment at 3.8 kV/cm and 10 kJ/kg increased total phenolics, flavonoids, and antioxidant activity by 8%, 31%, and 36%, respectively, while allowing comparable recovery with shorter extraction times or lower ethanol concentrations (Carpentieri et al. 2022). Its main benefit may therefore be reduced solvent use and processing time, particularly for wet cranberry pomace processed directly after juicing. Cranberry‐specific studies should assess the effects on A‐type PAC recovery and polymerization. In rehydrated jaboticaba pomace, PEF also altered phenolic‐polysaccharide associations, indicating matrix effects beyond membrane electroporation (Bocker and Silva 2026).

Sequential fractionation may provide another route. In blueberry pomace, Capaldi et al. (2024) first used citric‐acidified water at 80°C to recover an anthocyanin‐rich fraction, followed by subcritical water at 150°C to obtain a phenolic‐rich second fraction. A similar sequence could separate thermolabile cranberry anthocyanins before extracting less accessible constituents. However, the higher‐temperature step may degrade residual anthocyanins or alter PACs, requiring compound‐specific analysis. Its laboratory scale and 30:1 liquid‐to‐solid ratio also raise scale‐up concerns.

Industrial feasibility depends on the full processing chain, not extraction yield alone. Laboratory workflows often require drying, milling, high liquid‐to‐solid ratios, centrifugation, evaporation, and freeze‐drying. In a life‐cycle assessment, the cherry press‐cake pathway had the highest environmental impact, with electricity contributing at least 93% of the burden and evaporation plus repeated freeze‐drying accounting for approximately 80% of electricity use (Carpentieri et al. 2025). Cranberry processes should therefore prioritize wet‐pomace treatment, higher solids loading, solvent recovery, and less energy‐intensive concentration or drying, while additional fractionation should be justified by product yield and value.

Commercial feasibility is also constrained by process economics. For PEF‐assisted ethanol–water extraction of grape pomace, Soltanipour et al. (2025) reported a 2.72% reduction in unit production cost and an 8.11% increase in return on investment relative to conventional extraction. However, profitability depended mainly on extract price, ethanol use, and solid‐to‐liquid ratio rather than PEF equipment cost. Because the model assumed near‐continuous operation, commercial cranberry processing would also need to address seasonal pomace availability through storage, stabilization, or processing of additional feedstocks.

Chokeberry pomace further shows that circularity does not guarantee commercial viability. Agraso‐Otero et al. (2025) found that biorefineries combining phenolic recovery with either bioethanol and lignin production or anaerobic digestion were not profitable at the scales assessed, although the bioenergy route had a lower environmental impact, and steam production remained the main hotspot. Greater process complexity may therefore increase environmental and economic burdens unless product yields and market value offset the additional costs.

To date, no cranberry‐pomace study has combined pilot‐scale extraction with comprehensive environmental and economic assessments. Future work should validate food‐grade processing of wet industrial pomace and quantify solvent recovery, energy and water use, downstream processing, product yield, and cost. Extracts should also be standardized using relevant markers, particularly anthocyanins and A‐type PACs, for clearly defined food or nutraceutical applications.

## 5 Bioactive Properties of Cranberry Pomace and Derived Fractions

### 5.1 Antioxidant and Anti‐Inflammatory Activity

**FIGURE 2:**
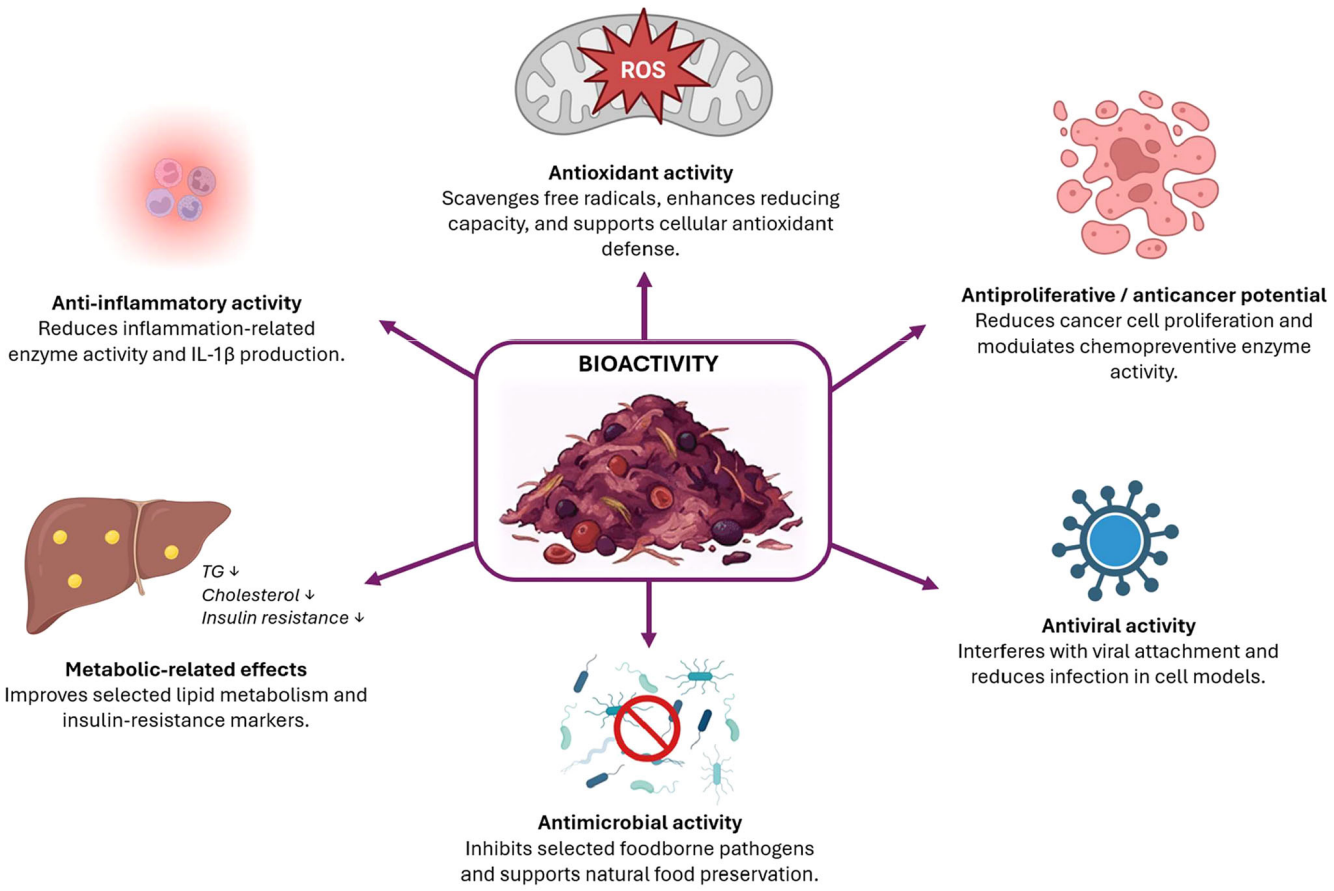
Main bioactive properties reported for cranberry pomace and derived fractions.

Antioxidant activity is among the most frequently reported properties of cranberry pomace and its derived fractions (Figure 2) (Ahmed et al. 2023; Viskelis et al. 2009; Otieno et al. 2026). This activity is associated with phenolic constituents retained after juice processing, including anthocyanins, flavonols, phenolic acids, and PACs, which can act as radical scavengers and reducing agents in chemical assays and may also influence cellular redox‐regulatory systems (Brzezowska et al. 2024; Parmar et al. 2016; White et al. 2010a). However, chemical antioxidant capacity should not be interpreted directly as evidence of physiological antioxidant effects.

In extracts from four American cranberry cultivars, radical‐scavenging activity ranged from 69.0% to 81.4% for whole berries and from 64.3% to 73.7% for the corresponding press cakes (Viskelis et al. 2009). The press cake extracts therefore retained substantial activity after juice removal. Nevertheless, their average radical‐scavenging activity was slightly lower than that of the berry extracts despite generally higher measured concentrations of phenolics and anthocyanins. This indicates that antioxidant‐assay responses were not determined solely by total phenolic concentration and may also have depended on phenolic composition, interactions among constituents, and extract characteristics. A recent solvent‐comparison study further demonstrated that the chemical antioxidant activity of cranberry PEs depends strongly on extract composition (Otieno et al. 2026). Among the six solvent systems, acetone–methanol–formic acid–water produced the highest total phenolic and PAC contents and the lowest half‐maximal inhibitory concentration (IC_50_) values in the DPPH, ABTS, and FRAP assays. Antioxidant activity correlated positively with total phenolics and PACs, whereas triterpenoid‐rich extracts showed weaker radical‐scavenging and reducing activity. These findings indicate that phenolic‐rich fractions, particularly those containing PACs, are the principal contributors to the chemical antioxidant responses measured, while also showing that extraction yield alone does not predict antioxidant potency.

Fractionation further demonstrated that different phenolic subclasses performed differently depending on the assay used. Parmar et al. (2016) found that the PAC‐rich A1 fraction exhibited the greatest reducing capacity, with a FRAP value of 2609.4 µmol TE/g DW, whereas flavonol‐ and anthocyanin‐rich fractions E4 and E5 showed particularly strong DPPH radical‐scavenging activity. The weak correspondence between the FRAP and DPPH results illustrates that reducing capacity and radical scavenging measure different chemical properties and should not be treated as interchangeable indicators of biological antioxidant efficacy.

More direct mechanistic evidence was obtained in an *in vitro* chicken hepatoma (LMH)‐cell model infected with *Salmonella enterica* serovar Enteritidis (Ahmed et al. 2023). Treatment with neutralized cranberry‐PE at 1 or 2 mg/mL improved the viability of infected LMH cells. At 6 h, the 2 mg/mL treatment increased the mRNA expression of Nrf2 and its associated antioxidant‐defense genes, heme oxygenase‐1 (HO‐1), thioredoxin (Txn), and Gclc, while the lower dose increased HO‐1, Txn, and Gclc without significantly increasing Nrf2. At 12 h, induction was maintained for several downstream genes but not for Nrf2. Nrf2 regulates cytoprotective systems involved in heme degradation, Txn‐dependent redox control, and glutathione synthesis through HO‐1, Txn, and Gclc, respectively. The results therefore support an Nrf2‐associated transcriptional response alongside improved cell survival. However, pathway activation was not confirmed through Nrf2 nuclear translocation, protein abundance, antioxidant enzyme activity, or direct measurements of oxidative stress, and the contribution of individual cranberry constituents was not established.

Anti‐inflammatory activity has also been reported for triterpenoid‐rich fractions obtained from an acetone extract of *V. macrocarpon* pomace (Mia 2026). The fractions inhibited lipoxygenase (LOX) and soluble epoxide hydrolase (sEH) to varying degrees and reduced lipopolysaccharide (LPS)‐induced interleukin‐1 beta (IL‐1β) production in human monocytic leukemia cell line (THP‐1) monocytes. Fraction F showed the strongest sEH inhibition, whereas fraction 2G produced comparatively modest LOX inhibition, with an IC_50_ of 104 µg/mL. The strongest suppression of IL‐1β was observed with Fraction D, which was rich in the neutral triterpenoids α‐ and β‐amyrin, while fractions containing ursolic acid, oleanolic acid, and their hydroxycinnamoyl esters also showed activity. Molecular docking provided complementary evidence for potential interactions between these constituents and inflammation‐related targets. Oleanolic acid showed the most favorable predicted interaction with sEH, with a YASARA interaction score of 11.6 kcal/mol, followed by α‐amyrin, ursolic acid, and corosolic acid. Favorable interactions were also predicted between several triterpenoids and phytosterols and the inflammasome‐associated proteins NOD‐like receptor family pyrin domain‐containing protein 3 (NLRP3) and caspase‐1. These findings suggest that the lipophilic fraction of cranberry pomace may influence inflammatory responses through enzyme inhibition and suppression of IL‐1β production. However, the docking results represent predicted binding rather than demonstrated target inhibition, and direct regulation of NLRP3 or caspase‐1 was not experimentally measured.

Cranberry pomace exhibits reducing and radical‐scavenging capacity in chemical assays, while limited cell‐based evidence indicates modulation of antioxidant‐defense genes and inflammatory responses. These findings remain predominantly *in vitro*, and physiological antioxidant or anti‐inflammatory effects have not been demonstrated in animal or human studies specifically examining cranberry pomace.

### 5.2 Chemopreventive and Antiproliferative Activity

Anticancer‐related activity has been reported for cranberry pomace and its derived fractions, although the evidence remains dominated by *in vitro* studies. Investigations have focused mainly on the induction of quinone reductase (QR/NQO1) as a marker of Phase II detoxification, inhibition of tumor cell proliferation and clonogenic growth, and, in a limited number of studies, disruption of cell cycle progression and induction of apoptosis (Caillet et al. 2011; Caillet, Lorenzo, Côté, Doyon, et al. 2012; Vu et al. 2012; Parmar et al. 2016; Tamkutė, Jančiukė, et al. 2022; Ferguson et al. 2004). Limited animal evidence has also shown reduced growth of U87 xenografts following administration of a press cake‐derived flavonoid fraction (Ferguson et al. 2006). Phenolic acids, flavonols, anthocyanins, flavan‐3‐ols, PACs, and pentacyclic triterpenoids have each been associated with these effects; however, no single class has been established as responsible, and activity varies with extract composition, concentration, and experimental model (Caillet et al. 2011; Ferguson et al. 2004; Ferguson et al. 2006; Mia 2026).

An early indication of chemopreventive potential was reported for *V. macrocarpon* PEs in Hepa 1c1c7 murine hepatoma cells, where induction of QR, also known as NAD(P)H:quinone oxidoreductase 1 (QR/NQO1), was used as a marker of Phase II detoxification activity (Caillet et al. 2011). NQO1 catalyzes the two‐electron reduction of electrophilic quinones to more stable hydroquinones, thereby limiting redox cycling and the formation of reactive intermediates. The water‐soluble, apolar, and anthocyanin‐rich PEs induced QR to varying degrees, although their responses were generally weaker than those of the corresponding fruit and earlier processing fractions. Moreover, the anthocyanin‐rich PE inhibited QR at higher concentrations. None of the samples produced detectable growth inhibition in Hepa 1c1c7 cells, with IC_50_ values above 200 mg/mL. These findings therefore demonstrate retained, but comparatively limited and concentration‐dependent, Phase II enzyme‐inducing activity in cranberry pomace rather than greater activity than the fruit.

This concept was examined in greater detail using polarity‐based HPLC fractions of cranberry fruit and PEs (Caillet, Lorenzo, Côté, Doyon, et al. 2012). QR‐inducing activity varied markedly with fraction polarity, composition, and concentration. Among the pomace‐derived fractions, the strongest response was produced by the least polar, PAC‐enriched fraction of the apolar phenolic extract, which reached 11.89 II(QR)/mg fraction at 25 mg/mL. Within the anthocyanin‐rich PE, the most polar fraction reached 10.74 II(QR)/mg fraction, whereas the least polar fraction showed substantially lower maximum activity of 2.65 II(QR)/mg fraction and inhibited QR at higher concentrations. Across the cranberry samples examined, fractions enriched in phenolic acids or PACs generally produced stronger QR induction than intermediate‐polarity fractions, while no detectable cytotoxicity was observed, with IC_50_ values above 50 mg/mL. These findings indicate that the Phase II enzyme‐inducing activity of cranberry pomace depends on the composition and concentration of the recovered fraction rather than on the total phenolic content alone.

More direct evidence of antiproliferative mechanisms was provided by Ferguson et al. (2004), who fractionated a warm‐water extract of *V. macrocarpon* press cake and identified a flavonoid‐rich fraction (Fr6) with activity against eight human tumor cell lines. In MDA‐MB‐435 cells, Fr6 produced dose‐dependent accumulation in the G2/M phase, reduced the proportion of cells in S phase, and increased annexin V‐positive cells, indicating that growth inhibition involved both disruption of cell cycle progression and induction of apoptosis. However, the fraction remained chemically heterogeneous, and the individual compounds responsible for these effects were not established. The same press cake‐derived fraction was subsequently evaluated in an animal model (Ferguson et al. 2006). Repeated intraperitoneal administration of Fr6 slowed the growth of U87 glioblastoma xenografts in immunocompromised mice, while complementary *in vitro* experiments showed transient G1 accumulation and increased cell death in U87 cells. This provides limited animal evidence for anticancer‐related activity of a cranberry‐pomace fraction. Nevertheless, the non‐oral route of administration, relatively high dose, treatment‐associated weight loss, and chemically undefined nature of the fraction limit direct translation to dietary or functional food applications. In the same study, inhibition of HT‐29 and DU145 xenografts was produced by a PAC‐rich fraction isolated from whole cranberries rather than pomace and therefore should not be attributed to the processing coproduct.

Antiproliferative activity was also reported for fractionated *V. macrocarpon* PEs in HepG2 human hepatocellular carcinoma cells (Parmar et al. 2016). The strongest effects were observed for the more polar ethanol fractions. Fractions E1 and E2, enriched in phenolic acids and flavan‐3‐ol monomers, and fraction E3, containing flavonols and anthocyanins, showed the lowest IC_50_ values of 7.8, 15.8, and 20.1 mg/L, respectively. At 100 mg/L, E2 reduced viable‐cell metabolic activity by more than 85%, while E1 and E2 produced at least 50% inhibition at lower concentrations. In contrast, the less active fractions showed IC_50_ values of approximately 62–94 mg/L. These findings suggest that activity was influenced more by fraction composition than by total phenolic content alone, with phenolic acids and monomeric flavan‐3‐ols showing greater potency in this model than several flavonol‐, anthocyanin‐, or PAC‐rich fractions. However, the study relied on a 3‐(4,5‐dimethylthiazol‐2‐yl)‐5‐(3‐carboxymethoxyphenyl)‐2‐(4‐sulfophenyl)‐2*H*‐tetrazolium (MTS) viability assay and did not examine apoptosis, cell cycle regulation, or specific signaling pathways; the observed response therefore demonstrates reduced viable‐cell metabolic activity rather than a defined anticancer mechanism. Comparable findings have been reported for biotransformed grape PEs, in which catechin and epicatechin were the predominant quantified flavan‐3‐ols. These chemically complex extracts reduced the growth of Caco‐2 and SW620 colorectal cancer cells and altered the cell cycle distribution, particularly in SW620 cells (Mišković Špoljarić et al. 2023). This comparison supports the broader relevance of flavan‐3‐ol‐rich pomace fractions to antiproliferative activity, although the effects cannot be attributed specifically to catechin, epicatechin, or other individual constituents.

Related observations were reported for cranberry processing extracts tested against HT‐29 and LS‐513 colon cancer cells (Vu et al. 2012). All tested extracts and juices reduced viable‐cell metabolic activity, with IC_50_ values ranging from 3.8 to 179.2 µg GAE/mL, although potency varied considerably with the processing stage, phenolic fraction, pH, and cell line. The anthocyanin‐rich PE was the least active preparation against both cell lines, whereas several water‐soluble and apolar phenolic extracts obtained from fruit or earlier processing fractions showed greater potency. Because the study relied on a water‐soluble tetrazolium salt‐1 (WST‐1) assay, these results demonstrate reduced viable‐cell metabolic activity but do not identify whether the response involved growth arrest, apoptosis, or another mechanism. Nevertheless, they confirm that pomace‐derived fractions retain measurable activity against colorectal cancer cells. A comparison with other berry pomaces further illustrates that such effects cannot be predicted from total phenolic content or antioxidant capacity alone. Stanca et al. (2024) found that blueberry PEs produced stronger effects on C2BBe1 cell viability and proliferation than chokeberry extracts, despite their lower total polyphenol content and antioxidant activity. The comparatively weak response of anthocyanin‐rich cranberry pomace fractions should therefore be interpreted as reflecting differences in phenolic composition, extract characteristics, and cellular sensitivity rather than simply lower phenolic abundance.

Antiproliferative effects were also evaluated using pressurized ethanol extracts recovered from supercritical‐CO_2_‐defatted *V. oxycoccos* pomace (Tamkutė, Jančiukė, et al. 2022). The cranberry extract was tested at 40, 200, and 1000 µg/mL against HCT116 and DLD1 colorectal carcinoma cells. HCT116 cells were more responsive than DLD1 cells, although the effects of the cranberry extract were modest compared with the corresponding chokeberry extract. At 1000 µg/mL, more than 69% of HCT116 cells remained viable after cranberry extract treatment, while no clear reduction in DLD1 viability was observed; in contrast, chokeberry extract reduced viability to approximately 51% and 50% in HCT116 and DLD1 cells, respectively. Flow cytometric analysis similarly showed only minor changes in viable and apoptotic cell fractions following cranberry extract treatment. More apparent effects emerged during prolonged clonogenic exposure: cranberry extract reduced the HCT116 colony area at 1000 µg/mL and decreased DLD1 colony formation, including reductions in colony number at lower concentrations and in colony size at 1000 µg/mL. These findings indicate modest, cell line‐ and assay‐dependent effects that were more evident during long‐term clonogenic growth than in short‐term viability assays, while providing no evidence for a defined molecular mechanism.

Recent dissertation work extended the evidence to lipophilic fractions of *V. macrocarpon* pomace enriched in pentacyclic triterpenoids (Mia 2026). The crude acetone extract produced a comparatively modest reduction in HT‐29 cell metabolic viability, with an IC_50_ of approximately 113 µg/mL after 24 h. Fractionation substantially increased activity, with Fractions 2F, 2G, and 2K showing IC_50_ values of 12–17 µg/mL. These fractions contained predominantly ursolic acid, oleanolic acid, and their hydroxycinnamoyl esters. Annexin V/propidium iodide flow cytometry at concentrations close to the IC_50_ further showed increased early‐ and late‐apoptotic cell populations, indicating that the reduction in viable cells involved the induction of apoptosis. The evidence broadens the bioactive profile of cranberry pomace beyond its phenolic constituents and identifies triterpenoid‐rich fractions as another source of antiproliferative activity. However, the fractions remained chemically complex, and neither caspase activation nor other apoptosis‐related signaling proteins were measured experimentally; the effects therefore cannot be attributed conclusively to individual triterpenoids or a specific molecular pathway.

Cranberry pomace exhibits anticancer‐related activity through induction of QR/NQO1 as a marker of phase II detoxification, reduction of viable tumor cell growth, disruption of cell cycle progression, induction of apoptosis, and inhibition of clonogenic or xenograft growth. The magnitude and nature of these effects depend strongly on the extract composition, concentration, and experimental model. Fractions enriched in phenolic acids, flavan‐3‐ols, PACs, or pentacyclic triterpenoids frequently showed greater activity than anthocyanin‐rich preparations, although comparisons among studies are limited by differences in extraction, fraction composition, concentration, and experimental model. Evidence remains predominantly *in vitro*, with limited animal data and no human studies specifically examining cranberry pomace. The contribution of its characteristic A‐type PACs relative to other retained phenolics therefore remains unresolved.

### 5.3 Metabolic‐Related Effects

The metabolic‐related effects of cranberry pomace have been examined in only a small number of animal studies, with evidence largely limited to diet‐induced metabolic disturbances. In growing Sprague–Dawley rats fed a high‐fructose diet, supplementation with 3% extruded cranberry pomace (ECP) partially attenuated several adverse metabolic changes (Khanal et al. 2010). Relative to the high‐fructose control, fasting insulin decreased by approximately 40%, total cholesterol by 37%, triacylglycerols by 34%, and homeostatic model assessment of insulin resistance (HOMA‐IR) by 42%. Fasting glucose and oral glucose tolerance were not significantly affected, indicating that the response was more apparent in insulin demand and lipid‐related indices than in glycemic control. These findings support a selective effect of cranberry pomace on insulin resistance and circulating lipid markers under high‐fructose feeding, although the study did not establish the underlying molecular mechanisms. Comparable effects have been reported for other berry pomaces in closely related diet‐induced models. In high‐fructose‐fed growing Sprague–Dawley rats, blueberry pomace included at 3% of the diet reduced fasting insulin and HOMA‐IR to values comparable with the control group while also lowering fasting triacylglycerols, plasma cholesterol, and total abdominal fat relative to the high‐fructose group (Khanal et al. 2012). These effects were observed with both unextruded and extruded preparations, with no consistent additional benefit from extrusion. As with cranberry pomace, fasting glucose and oral glucose tolerance were unchanged, indicating a clearer effect on insulin demand and lipid‐related endpoints than on glycemic control. Postprandial triacylglycerols were also not significantly reduced, showing that the metabolic response was selective rather than uniformly lipid‐lowering.

Related findings were reported in Wistar rats fed a high‐fat diet for 8 weeks and supplemented with 3% unextruded cranberry pomace (UCP) or 3% ECP (Bajerska et al. 2018). Serum triacylglycerols decreased from 102.3 mg/dL in the high‐fat control group to 70.2 mg/dL with UCP and 87.4 mg/dL with ECP, although only the reduction produced by UCP was statistically significant. Total cholesterol declined from 117.7 mg/dL to 103.4 and 103.2 mg/dL with UCP and ECP, respectively, but neither change reached statistical significance. UCP also significantly increased fecal lipid excretion and reduced the liver index, whereas ECP did not differ significantly from the high‐fat control for these endpoints. Both preparations increased plasma FRAP and GSH and reduced thiobarbituric acid reactive substances (TBARS), despite extrusion substantially decreasing anthocyanins, flavonols, and tocochromanols in the pomace. These findings support selective effects of cranberry pomace on lipid handling and oxidative status under high‐fat feeding, while also showing that extrusion did not improve metabolic efficacy in this model and weakened some responses observed with the unextruded preparation.

Further support for a combined fiber–polyphenol contribution comes from studies of strawberry and blackcurrant pomaces. In fructose‐fed rats, a polyphenol‐rich strawberry pomace preparation produced greater reductions in serum insulin, HOMA‐IR, hepatic total cholesterol, hepatic conjugated dienes, and serum free fatty acids than a preparation from which most polyphenols had been removed (Jaroslawska et al. 2011). The polyphenol‐reduced preparation nevertheless retained some metabolic activity and still contained residual phenolics, so the findings support contributions from both the fiber matrix and retained polyphenols rather than allowing their effects to be separated completely. Similarly, polyphenol‐rich fiber preparations derived from blackcurrant and strawberry pomace reduced epididymal fat mass, prevented high‐fat‐diet‐induced hyperinsulinemia, lowered cholesterol‐related markers, and restored cecal short‐chain fatty acid formation when administered throughout the 8‐week feeding period; delayed supplementation during only the final 4 weeks was generally less effective (Jurgoński et al. 2016). These comparisons support the interpretation of berry pomaces as complex fiber–phenolic matrices, although the intestinal fermentation and component‐specific mechanisms demonstrated in these studies have not been established directly for cranberry pomace.

The animal studies on cranberry pomace indicate more consistent effects on triacylglycerols, cholesterol‐related markers, insulin‐related indices, fecal lipid excretion, and oxidative status than on fasting glucose or glucose tolerance. Its metabolic effects therefore appear to involve lipid handling and insulin demand rather than a direct hypoglycemic action. Comparisons with other berry pomaces suggest that interactions between dietary fiber and retained phenolics may also contribute, although intestinal fermentation has not been measured directly for cranberry pomace. Processing influenced the response in a model‐dependent manner. Extrusion produced the clearest effects under high‐fructose feeding, whereas the unextruded preparation was more effective for several endpoints under high‐fat feeding. These contrasting findings show that the effect of extrusion cannot be generalized across dietary models and should be interpreted in relation to the accompanying changes in phenolic composition and fiber structure.

### 5.4 Antiviral Activity

Antiviral activity has also been reported for cranberry pomace, although evidence remains limited. Cranberry PE inhibited Zika virus (ZIKV) and dengue virus serotypes 2 and 4 (DENV‐2 and DENV‐4) in A549 lung epithelial and Huh7.5 hepatoma cells (Tamkutė, Haddad, et al. 2022). The extract reduced viral infection with relatively low host‐cell cytotoxicity, showing IC_50_ values of 54.2 µg/mL for DENV‐2 and 40.3 µg/mL for DENV‐4, compared with CC_50_ values of 278.2 µg/mL in A549 cells and 425.0 µg/mL in Huh7.5 cells. Mechanistic observations suggested a direct interaction with viral particles and interference with host‐cell attachment. The extract was also well tolerated in adult zebrafish at 400 µg/g body weight.

This mode of action is consistent with broader evidence for the antiflaviviral activity of polyphenols. Vázquez‐Calvo et al. (2017) showed that delphinidin and epigallocatechin gallate inhibited West Nile virus mainly by disrupting early attachment and entry and exerting direct virucidal effects. The same compounds also reduced ZIKV and DENV‐2 infectivity, indicating that selected polyphenols can act against several flaviviruses. Because cranberry pomace contains anthocyanins, flavonols, and PACs, its antiviral effect may similarly involve interactions with viral particles or early infection events. However, compared with its antioxidant, antimicrobial, and antiproliferative properties, the antiviral evidence remains preliminary.

## 6 Food Applications of Cranberry Pomace

### 6.1 Cranberry Pomace as a Functional Ingredient in Foods

**FIGURE 3:**
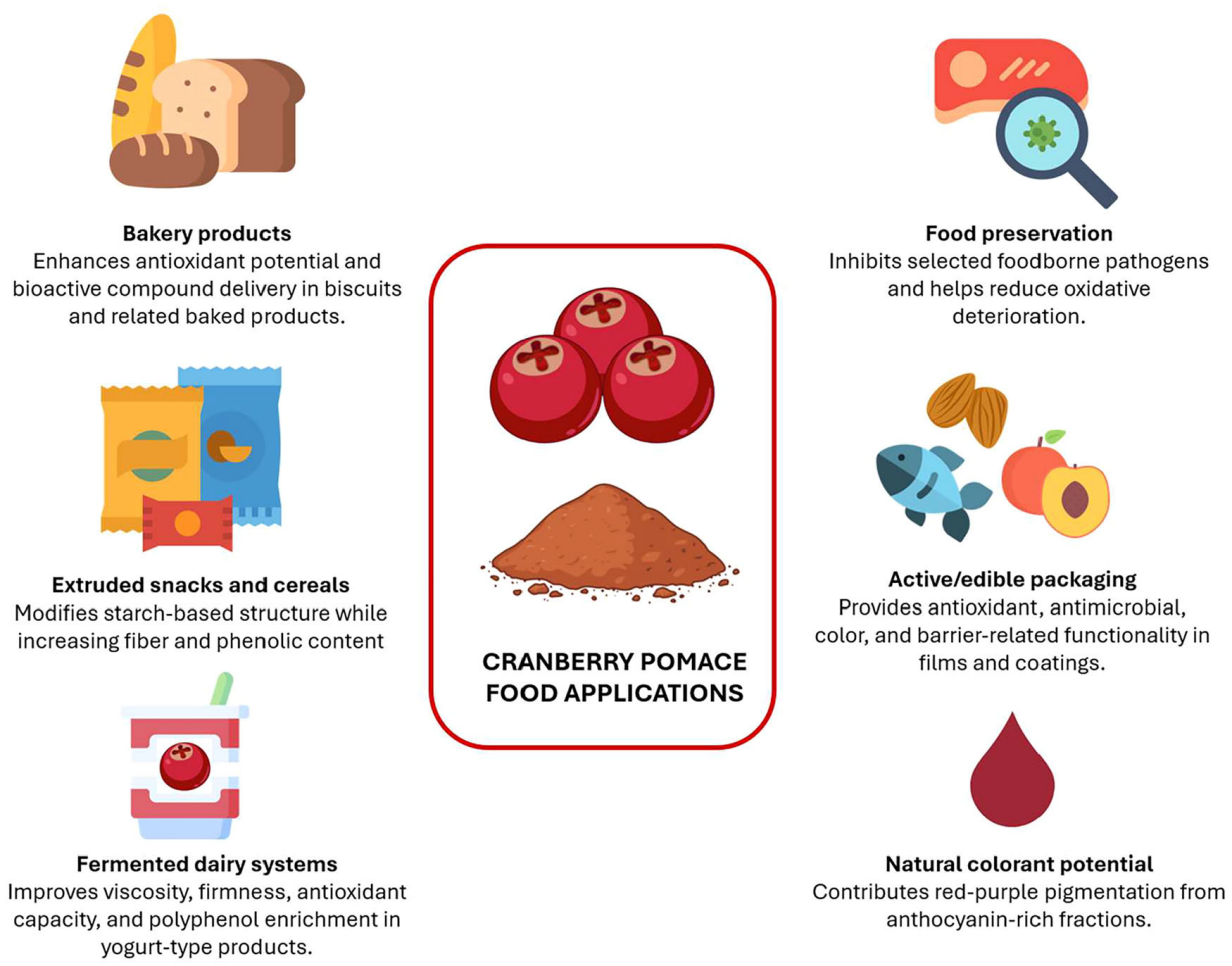
Food applications of cranberry pomace and derived ingredients.

Cranberry pomace has attracted attention as a multifunctional food ingredient because it retains dietary fiber, phenolic constituents, and natural pigments after juice processing. These components may improve the compositional value of foods while also influencing hydration, texture, color, and matrix structure. Therefore, cranberry pomace offers potential for value‐added utilization in foods with improved functional and technological attributes (Figure 3).

Extrusion‐based products represent one of the most frequently investigated routes for the utilization of cranberry pomace. Early work with cranberry (*V. macrocarpon*) pomace–corn starch extrudates containing 30%–50% pomace showed that extrusion altered the phenolic profile, with decreases in anthocyanins and flavonols but concomitant increases in procyanidin extractability and oxygen radical absorbance capacity (ORAC) values relative to the corresponding unprocessed blends (White et al. 2010a). These changes were attributed to the release of matrix‐associated phenolics and the formation of lower molecular‐weight compounds during extrusion, suggesting that processing may improve the accessibility of selected pomace constituents. Similarly, incorporation of cranberry pomace into corn starch extrudates at 50, 150, and 300 g/kg markedly affected the product structure, with increasing pomace levels reducing the sectional expansion index and specific length, while increasing the piece density and water solubility index (S. Wang et al. 2019). Such effects were mainly associated with the high fiber content of pomace and its influence on starch matrix development during extrusion.

Further mechanistic insight was provided by Richter et al. (2022), who showed that cranberry pomace altered starch transformation and hydration behavior during extrusion, thereby affecting the expansion and textural development of direct‐expanded products. In particular, pomace addition reduced radial expansion and modified sectional mechanical properties, while also changing water absorption and solubility behavior. These observations indicate that cranberry pomace acts not only as a passive fortifying ingredient, but also as a matrix‐modifying component capable of influencing starch gelatinization, melt behavior, and final product structure. Additional work has shown that cranberry pomace can also be incorporated successfully into protein‐rich extruded snacks (Acharya 2020), deep‐fat fried extruded snacks (Bernin 2022), and breakfast cereal extrudates (Bickelhaupt 2022), with formulations generally containing approximately 5%–15% pomace while maintaining acceptable product quality. In addition, enzymatic treatment of cranberry pomace has been shown to improve water‐holding, oil‐holding, swelling, and emulsion‐stabilizing properties while also enhancing prebiotic potential, which further supports its suitability as a multifunctional food ingredient (Jagelaviciute et al. 2022).

Sensory evaluation of corn‐based puffed snacks containing 5%–25% cranberry pomace showed that acceptance depended strongly on the level of incorporation (Kloepfer 2022). The formulation containing 5% pomace was the most accepted pomace‐enriched product, while samples containing 5%–15% retained texture‐related liking broadly comparable with the control. At 20%–25% incorporation, consumer acceptance declined as cranberry color and flavor, sourness, bitterness, astringency, density, and chewiness became more pronounced. These findings suggest that moderate inclusion levels may preserve acceptable sensory quality, whereas higher substitution levels intensify both flavor‐related and structural limitations.

Bakery applications have also been examined. In biscuits fortified with 5%, 10%, and 15% pomace from American cranberry , replacement of wheat flour with cranberry pomace increased the total polyphenol content, antioxidant activity, and bioactivity of digested fractions (Matłok et al. 2025). The digested biscuit extracts reduced intracellular reactive oxygen species (ROS) formation in *Saccharomyces cerevisiae* exposed to oxidative stress and inhibited selected enzymes, including acetylcholinesterase (AChE), cyclooxygenase‐1 (COX‐1), and cyclooxygenase‐2 (COX‐2), with the strongest effects observed at 5 mg/mL. These findings indicate that cranberry pomace can function not only as a compositional enhancer in bakery products, but also as a source of bioaccessible constituents capable of retaining biological activity after digestion. A similar pattern has been reported for raspberry pomace in cereal‐based products, where partial replacement of flour increased dietary fiber and phenolic content but also altered dough handling, texture, and product structure because of water binding and insoluble particles (Ali Redha and Esatbeyoglu 2026). This comparison suggests that cranberry pomace, like other berry pomaces, should be treated as a matrix‐active ingredient rather than only as a phenolic fortifier.

Dairy applications have been explored mainly in yogurt systems, where cranberry pomace has been used both as a fiber‐rich particulate ingredient and as a polyphenol‐rich extract. The addition of 2.0%–4.5% dietary fiber‐rich pomace from cranberry (*V. macrocarpon*) increased total phenolic content and antioxidant capacity in a dose‐dependent manner, improved firmness and viscosity, reduced whey separation, and maintained yogurt culture viability; moreover, the bioaccessibility index of polyphenols after intestinal digestion was approximately 90% (Varnaitė et al. 2022). The timing of pomace incorporation was also important because addition before fermentation led to lower post‐gastric and post‐intestinal protein hydrolysis than addition after fermentation, indicating stronger interactions between pomace constituents and the dairy matrix. A related study using 0.25%–0.75% cranberry PE showed that protein–polyphenol interactions accelerated acid‐induced gelation, promoted the formation of larger aggregated protein networks, increased viscosity, and lowered syneresis, while viable lactic acid bacteria remained slightly above 10^9^ colony‐forming units (CFU)/g during refrigerated storage (Zygmantaitė et al. 2021). However, only 18.2%–21.7% of the initial phenols were recovered in gastrointestinal fluids at the end of digestion, suggesting that although cranberry pomace‐derived ingredients can improve the technological properties of yogurt, their phenolic release may remain limited by interactions with milk proteins. More recently, smoothies containing enzymatically modified cranberry pomace were perceived as thicker, more acidic, and less sweet than the control (Jagelavičiūtė et al. 2026). However, the formulation containing Celluclast 1.5 L‐treated pomace received the most favorable ratings for color, cranberry taste, acidity, and overall acceptability. This indicates that sensory performance depends not only on the amount of pomace incorporated but also on its pretreatment and resulting physicochemical properties.

Cranberry pomace is a multifunctional, matrix‐active ingredient whose effects depend on dose, processing, ingredient form, and food composition. In extruded cereals, it provides fiber and phenolics but may reduce expansion and alter texture. In bakery products, it can enhance polyphenol content and digestion‐related bioactivity, although higher inclusion may impair dough handling and sensory quality. In yogurt, pomace and its extracts can improve viscosity and firmness, reduce syneresis, and maintain culture viability, but protein–polyphenol interactions may restrict phenolic release during digestion.

### 6.2 Food Preservation and Antimicrobial Applications

Cranberry pomace has been investigated as a natural preservative ingredient, particularly in meat systems where microbial growth and lipid oxidation occur simultaneously. Its preservative value is mainly linked to phenolic constituents that inhibit foodborne pathogens and spoilage microorganisms while also contributing to antioxidant protection (Figure 3).

Mechanistic support was provided by American cranberry PEs and fractions tested against *S. enterica* serovars, where the ethyl acetate fraction showed the strongest inhibition, with minimum inhibitory concentration (MIC) values of 0.45–1.80 mg/mL depending on the fraction and serovar (Das et al. 2019). The extracts also downregulated genes related to motility, invasion, and stress survival, indicating effects beyond simple growth inhibition. Earlier work with solid‐state fermented cranberry pomace similarly showed inhibition of *Listeria monocytogenes*, *Vibrio parahaemolyticus*, and *Escherichia coli* O157:H7, with enhanced activity attributed to increased phenolic release during fermentation (Vattem et al. 2004). These observations support the view that cranberry pomace may serve as a source of food‐grade antimicrobial constituents with broad‐spectrum activity.

Direct validation in meat systems showed that American and swamp cranberry PEs limited pathogen growth in minced pork and pork burgers during refrigerated storage, with stronger effects against *L. monocytogenes* and *S*. Enteritidis than *E. coli* (Gniewosz and Stobnicka 2017; Stobnicka and Gniewosz 2018). Fruit extracts were generally more active than PEs, but neither preparation impaired sensory acceptability. In fermented meat systems, cranberry pomace ethanol extract inhibited *E. coli* O157:H7, *S*. Enteritidis, and *L. monocytogenes* while having less effect on selected starter cultures, supporting pathogen control without disrupting fermentation (Lau et al. 2019). In pork slurry, burgers, and cooked ham, ethanolic extracts were also more effective than aqueous extracts in limiting microbial growth and oxidative deterioration, indicating dual antimicrobial and antioxidant functionality (Tamkutė et al. 2019).

In preservation systems, cranberry PEs show clear potential as natural antimicrobial agents, particularly in meat and meat‐based products. Their efficacy appears to be greatest against Gram‐positive bacteria, especially *L. monocytogenes*, which may be attributable, at least in part, to differences in cell envelope structure because the outer membrane of Gram‐negative bacteria can restrict the penetration of phenolic compounds and thereby reduce susceptibility. In addition, cranberry PEs may provide dual functionality by contributing both antimicrobial and antioxidant effects, which is advantageous for the preservation of highly perishable foods.

### 6.3 Packaging Applications

Cranberry pomace has been used in edible films, bioactive coatings, and active packaging. Its fiber‐ and pectin‐rich matrix may contribute to film formation, while phenolics and pigments provide antioxidant, antimicrobial, and color‐related functions. It can therefore serve either as a structural packaging component or as a source of bioactive extracts incorporated into films and coatings.

One of the earliest demonstrations of this potential was reported by Park and Zhao (2006), who developed pectin–plasticizer films containing *V. macrocarpon* pomace water extract. Depending on the formulation, the tensile strength was 8.9–11.6 MPa, elongation at break was 3.9%–10.2%, and water vapor permeability was 2.2–4.0 × 10^−10^ g m/m^2^ s Pa, indicating useful mechanical and barrier properties but limited flexibility. Similar trade‐offs were observed in blackcurrant pomace films, which gained antioxidant and pH‐sensitive color functions but showed weaker mechanical performance and, in some formulations, greater water vapor permeability (Kurek et al. 2021). Polymer optimization is therefore essential to balance active functionality with film integrity.

This concept was extended to almond coatings containing cranberry PE in pectin or pectin–beeswax matrices (Rodriguez Martinez 2017). The extract supplied anthocyanins and other phenolics, while the coating reduced peroxide formation and improved oxidative stability during storage, supporting its use as an antioxidant component for lipid‐rich foods. Comparable blackberry pomace coatings also provided antioxidant and moderate antimicrobial activity, although performance depended strongly on matrix composition, with bacterial cellulose improving water–vapor resistance and pectin affecting viscosity and dissolution (Isopencu et al. 2021).

Cranberry PE has also been incorporated into hydroxypropyl methylcellulose (HPMC)‐based edible separation sheets for fruit leather (Jung et al. 2024). The extract supplied natural color, flavor, and phenolics, while a cellulose‐nanocrystal Pickering emulsion reduced water‐vapor permeability from approximately 0.59 to 0.033 g mm/m^2^ day Pa. Thus, sensory and bioactive functions were provided by the extract, whereas barrier improvement depended mainly on matrix engineering. Similar matrix‐dependent release was observed in blueberry pomace films, where active compounds migrated more readily into acidic aqueous solutions than fatty simulants (Singh et al. 2020).

Cranberry pomace‐derived films and coatings have also shown direct preservative effects. A pectin coating from cranberry pomace extended peach shelf life at room temperature from 4 to 8 days while reducing moisture loss and preserving firmness (F. Wang et al. 2025). Alginate–pectin films containing cranberry PE inhibited *L. monocytogenes* and *Pseudomonas aeruginosa* on herring fillets and reduced TBARS, total volatile nitrogen (TVN), and biogenic amine accumulation during storage (Urbonavičiūtė et al. 2023). These systems therefore offer both physical and active protection against microbial and oxidative spoilage.

Cranberry pomace can serve both as a structural material and as a source of bioactive compounds in edible and active packaging. Similar to blackcurrant, blackberry, and blueberry pomace systems, improved antioxidant, antimicrobial, or color‐related functionality may be accompanied by changes in mechanical strength, water sensitivity, or barrier performance. Its effectiveness therefore depends on the film matrix, incorporation level, ingredient form, and target food.

## 7 Conclusions and Future Perspectives

**FIGURE 4:**
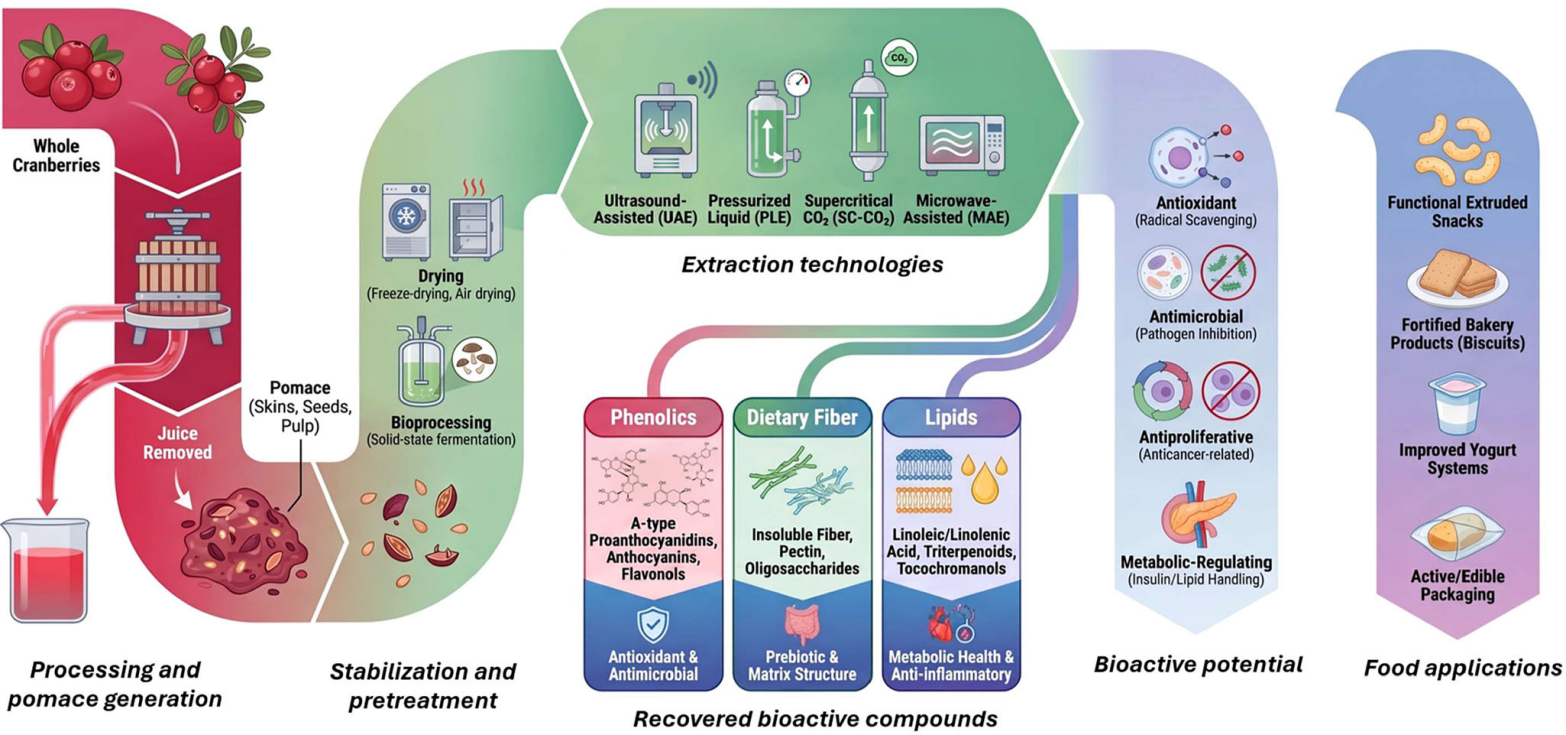
Schematic overview of cranberry pomace valorization, from pomace generation and pretreatment to bioactive compound recovery, associated health‐related activities, and potential food applications.

Cranberry pomace is a valuable coproduct containing phenolic, fiber‐rich, and lipophilic fractions with potential for food and ingredient applications. Ethanol extraction remains the practical benchmark, while ultrasound, PLE, and NADES offer further opportunities, subject to scalability, solvent recovery, energy demand, and cost. Antioxidant, antimicrobial, and antiproliferative effects are the most consistently reported, although evidence remains mainly preclinical. Applications in extruded, bakery, dairy‐type, and packaging systems further demonstrate its functional potential, as summarized in Figure 4.

Future work should now move beyond broad compositional descriptions toward a more functionally resolved understanding of cranberry pomace. The most immediate priority is to clarify the bioaccessibility, gastrointestinal transformation, and biological relevance of cranberry pomace polyphenols because their presence in the material does not necessarily indicate effective release, absorption, or physiological activity following consumption. To date, the release of matrix‐associated polyphenols during gastrointestinal digestion and their subsequent transformation into phenolic metabolites during colonic fermentation have not been systematically investigated. A second priority is to investigate, in cranberry pomace specifically, bioactivities that are more developed in the cranberry fruit literature but remain insufficiently examined for the pomace itself, particularly urinary tract‐related, cardiometabolic, neuroprotective, and hepatoprotective effects. Human intervention studies will also be required before the predominantly preclinical findings can support health‐related claims A third priority is the development of greener, more selective processing. Food‐grade NADES, enzyme‐assisted extraction, membrane fractionation, and cascade biorefinery approaches require further evaluation. Mild drying methods should also be compared with conventional drying for anthocyanin and A‐type PAC retention, energy efficiency, storage stability, and suitability for subsequent extraction or direct food use. Future studies should report cranberry species, cultivar, harvest and maturity details, production region, storage and pressing history, drying and extraction conditions, analytical method, calibration standard, and expression basis. Standardized reporting is essential for attributing compositional differences and enabling reliable cross‐study comparison.

Safety and quality control remain prerequisites for food‐grade use. Future studies should assess pesticide and heavy‐metal residues, microbial and mycotoxin contamination, and residual solvents, while establishing raw‐material specifications, hygienic stabilization, storage requirements, and batch‐to‐batch compositional consistency to support regulatory approval and reliable manufacture. Furthermore, emerging tools such as metabolomics, machine learning, and inline process monitoring may support compositional standardization and process optimization. Their value will depend on robust datasets and clearly defined applications within integrated cranberry‐pomace biorefinery systems.

Industrial translation is most realistic where cranberry pomace already provides demonstrated functions, particularly fiber enrichment, natural color, antioxidant protection, and antimicrobial activity. Near‐term applications therefore include bakery and snack products, fruit‐ and dairy‐based foods, and active or edible packaging. More fractionated nutraceuticals and multi‐stream biorefineries remain exploratory and require stronger evidence of techno‐economic feasibility, process robustness, safety, and regulatory acceptance. Progress will also depend on coordination among growers, processors, manufacturers, and packaging or nutraceutical sectors to match pomace fractions with appropriate end uses. Cranberry pomace should therefore be treated as a distinct valorization target.

## Author Contributions

**Ali Ali Redha**: conceptualization, investigation, writing – original draft, writing – review and editing, visualization. **Tuba Esatbeyoglu**: supervision, writing – review and editing, funding acquisition.

## Conflicts of Interest

The authors declare no conflicts of interest.

## Data Availability

The data presented in this study are available in the referenced articles.
